# Research Progress on perinatal *E. coli* infection

**DOI:** 10.3389/fmicb.2026.1840209

**Published:** 2026-06-03

**Authors:** Yuning Lin, Wenzhen Zhao, Hongyan Xie, Zhenyi Lv, Songping Chen, Hongwei Jin, Jianning Wu

**Affiliations:** 1 Medical Laboratory, Xiamen Humanity Hospital, Fujian Medical University, Xiamen, China; 2 Medical Laboratory, Zhangzhou Yuanshan Hospital, Zhangzhou, China

**Keywords:** antimicrobial resistance, *E. coli*, ExPEC, maternal colonization, neonatal sepsis, vaccine, vertical transmission, virulence factors

## Abstract

Against the backdrop of widespread implementation of intrapartum prophylaxis for group B streptococcus (*GBS*), *E. coli* has emerged as a major pathogen of early-onset neonatal sepsis (EOS) and neonatal meningitis. Vaginal colonization of pregnant women with highly virulent *E. coli* is not uncommon and has been associated with adverse obstetric outcomes, including preterm birth, chorioamnionitis, and neonatal infection. Available evidence suggests that the maternal colonization rate is approximately 20–30%, and vertical transmission during delivery occurs frequently. Colonizing strains often harbor virulence determinants associated with extraintestinal pathogenic *E. coli* (ExPEC), with virulence profiles resembling those of uropathogenic *E. coli* and neonatal meningitis-associated strains. Although most exposed neonates may remain asymptomatically colonized, once invasive infection occurs, the disease can rapidly progress to sepsis or meningitis, resulting in substantial mortality and long-term neurodevelopmental sequelae. In recent years, the spread of multidrug-resistant strains, particularly extended-spectrum *β*-lactamase (ESBL)-producing isolates, has further complicated perinatal prevention and empirical treatment. This review summarizes current advances in perinatal *E. coli* infection, with a focus on maternal colonization and vertical transmission, pathogenic mechanisms, clinical manifestations, antimicrobial resistance trends, and emerging preventive strategies.

## Introduction

*E. coli* is one of the major pathogens implicated in perinatal maternal and neonatal sepsis. It can initially colonize the maternal gastrointestinal and genitourinary tracts and may subsequently be transmitted from mother to infant during the perinatal period, leading to neonatal colonization and invasive infection (Tamelienė et al., 2012; Hwang et al., 2024; Flannery et al., 2022a). Recent studies have shown that among maternal perinatal colonizing bacteria, *E. coli* is one of the most commonly transmitted pathogens to neonates (Hwang et al., 2024; Shimeles et al., 2025). Molecular epidemiological studies have further shown that *E. coli* strains causing EOS share substantial similarity in phylogenetic characteristics and virulence profiles with maternal vaginal carriage strains identified in late pregnancy, thereby supporting a maternal vertical transmission origin (Malaure et al., 2024).

Within the pathogen spectrum of EOS, *E. coli* has become one of the leading Gram-negative pathogens, with a particularly prominent clinical burden in preterm infants and those with very low birth weight, and is associated with higher rates of severe illness and mortality (Stoll et al., 2020; Stocker et al., 2024; Hoffman et al., 2024). In addition, *E. coli*, together with GBS, constitutes the core pathogen spectrum of neonatal bacterial meningitis. In certain high-risk populations, particularly extremely preterm infants, the incidence of *E. coli* infection remains high and may even be increasing (Bauserman et al., 2013; Schrag et al., 2016; Miselli et al., 2022; Williams et al., 2021). Unlike GBS, for which routine antenatal screening and intrapartum antibiotic prophylaxis are recommended, no comparable routine pathogen-specific screening or preventive strategy currently exists for neonatal *E. coli* infection; meanwhile, neonatal *E. coli* colonization begins soon after birth (Tamelienė et al., 2012; Verani and Schrag, 2010; Diep et al., 2026). Meanwhile, neonatal sepsis remains one of the leading causes of neonatal morbidity and mortality worldwide, largely owing to the immaturity of the neonatal immune system (Kariniotaki et al., 2024; Raturi and Chandran, 2024). ExPEC represents the major pathogenic subgroup and can cause bacteremia, urinary tract infection, and neonatal meningitis (Poolman and Wacker, 2016; Sora et al., 2021; Aljohni et al., 2025). Clinical outcomes of neonatal *E. coli* infection are often severe, with high mortality and a substantial risk of long-term neurodevelopmental impairment among survivors (Thomas et al., 2024; de As et al., 2020; Nhu et al., 2024; Alkeskas et al., 2015).

Beyond the substantial disease burden, rising antimicrobial resistance has further complicated the clinical management of perinatal *E. coli* infection. In recent years, ESBL-producing *E. coli* has increased steadily and has been associated not only with a higher risk of perinatal maternal colonization and early-onset neonatal infection, but also with reduced effectiveness of empirical antimicrobial therapy, thereby markedly increasing the complexity of intensive care and antibiotic stewardship in the neonatal intensive care unit (NICU) (Fleischmann-Struzek et al., 2018; Choi et al., 2023b; Williams et al., 2022). *E. coli*-associated EOS typically develops within the first 1–3 days after birth and is usually characterized by nonspecific clinical manifestations, posing major challenges for early recognition and timely intervention (Kariniotaki et al., 2024; Celik et al., 2022; Lungu et al., 2024). More importantly, the global expansion of multidrug-resistant *E. coli*, particularly ESBL-producing isolates, has reduced the reliability of commonly used empirical agents, especially ampicillin and third-generation cephalosporins (Day et al., 2019; Flannery et al., 2021). In severe infections, this has contributed to greater use of carbapenems in clinical practice, raising further concern about the ongoing evolution of antimicrobial resistance (Tamma et al., 2024; Walker et al., 2024). This concern has also been reflected in pediatric studies of urinary tract infection and bloodstream infection, which show a rising ESBL burden and increasing challenges for empirical antibiotic selection (He et al., 2024; Park et al., 2022).

From an antimicrobial-resistance priority perspective, resistant *E. coli* should be considered within the broader Enterobacterales categories prioritized by major public-health agencies. The 2024 WHO Bacterial Priority Pathogens List classifies third-generation cephalosporin-resistant Enterobacterales and carbapenem-resistant Enterobacterales as critical-priority pathogens (WHO, 2024). Similarly, the CDC Antibiotic Resistance Threats Report classifies carbapenem-resistant Enterobacteriaceae as an urgent threat and ESBL-producing Enterobacteriaceae as a serious threat; both categories include *E. coli*. These classifications are particularly relevant to perinatal infection because ESBL-producing and multidrug-resistant *E. coli* may compromise empirical therapy for maternal and neonatal invasive infections (CDC, 2019).

Overall, with the continuing rise in the burden of ExPEC infection and antimicrobial resistance, perinatal *E. coli* infection has become a public health issue requiring urgent attention (Stoll et al., 2020; Meena et al., 2023). Taken together, the available evidence highlights the importance and urgency of further elucidating the patterns of maternal *E. coli* colonization, the routes of mother-to-infant vertical transmission, and the underlying molecular mechanisms of bacterial pathogenicity. Accordingly, this review systematically integrates the latest evidence on maternal colonization characteristics, transmission dynamics, virulence determinants, clinical phenotypes, antimicrobial resistance patterns, and emerging preventive strategies related to perinatal *E. coli* infection, with the aim of providing a comprehensive reference for both clinical practice and future research in this field.

## Epidemiological studies on *E. coli* colonization and mother-to-infant vertical transmission

Maternal genital tract colonization with *E. coli* is not uncommon during pregnancy. A meta-analysis including 82 cohort studies and 33,118 pregnant women reported an overall *E. coli* colonization rate of 29% (95% CI: 23–36%) (Moradi et al., 2021). Similarly, another study of 274 women in late pregnancy (gestational age ≥35 weeks; mean, 37.6 weeks) found an overall colonization rate of 34.7% for potential neonatal pathogens, with a vaginal *E. coli* colonization rate of 29.9% (82/274) (Birhane Fiseha et al., 2021). Prevalence estimates reported across different regions are summarized in Table 1. Current evidence suggests that vaginal *E. coli* colonization may partly originate from the maternal intestinal/rectal reservoir, followed by spread to the lower genital tract through perineal proximity (Liu et al., 2019; Amabebe and Anumba, 2020). At the same time, disruption of the vaginal microbiota may facilitate *E. coli* overgrowth and persistent colonization. In a healthy vaginal milieu, Lactobacillus spp. help maintain a pathogen-restrictive environment through lactic acid production, low pH, and other antimicrobial activities (Amabebe and Anumba, 2018). In pregnancy-associated vaginal microbiota, reductions in Lactobacillus abundance are accompanied by an increased representation of Enterobacterales, with *E. coli* showing the most pronounced increase in abundance in women with bacteriuria (Boutouchent et al., 2024). Importantly, however, protective activity differs among Lactobacillus species. *L. crispatus*, *L. jensenii*, and *L. gasseri* inhibit *E. coli* growth, whereas *L. iners* does not show the same inhibitory capacity, and this difference appears to be closely linked to pH and D-lactic acid production (Hudson et al., 2020). Consistent with this, *L. crispatus*-dominant vaginal communities are associated with stronger anti-*E. coli* activity, whereas *L. iners*, although frequently detected in the vaginal microbiota, is increasingly regarded as a transitional or less stable species that confers weaker protection against dysbiosis (Ghartey et al., 2014; Zheng et al., 2021). Earlier mechanistic work further showed that Lactobacillus-derived products can inhibit UPEC growth and attenuate adhesion-related virulence expression (Cadieux et al., 2009). Therefore, the microbial context favoring *E. coli* colonization is better described as a depletion of protective Lactobacillus species rather than a nonspecific loss of Lactobacillus spp. overall.

**Table 1:** Prevalence of maternal *E. coli* colonization during pregnancy.

Researcher (year)	Country/region	Research design	Sample size	Sampling points	Colonization rate of *E. coli*	Detection method	Ref.
Hwang et al. (2024)	South Korea	Retrospective cohort	173	Culture during labor (obtained from blood, urine and vagina during delivery)	30.6%	Culture-based method	Hwang et al. (2024)
Shimeles et al. (2025)	Ethiopia	Cross-sectional Study of Medical Institutions	348	Vaginal swab	27.6%	Culture-based method	Shimeles et al. (2025)
Moradi et al. (2021)	Global	Systematic review and meta-analysis	33,118	Vagina/Rectum	29%	culture-based+molecular methods	Moradi et al. (2021)
Birhane Fiseha et al. (2021)	Ethiopia	Ethiopia Cross-sectional study	274	Vaginal	29.90%	Culture-based method	Birhane Fiseha et al. (2021)
Liu et al. (2019)	Taiwan	Prospective Cohort	137	Vaginal-rectal	35.80%	Culture-based method+ multiplex PCR pathotype identification	Liu et al. (2019)
Saez-Lopez et al. (2016)	Spain	Prospective cohort	638	Vaginal	13%	Culture-based method+MALDI-TOF	Sáez-López et al. (2016b)
Steetskamp et al. (2024)	Germany	Retrospective cohort	327	Vaginal swab	22.9%	Culture-based method	Steetskamp et al. (2024)
Choi et al. (2023)	South Korea	Retrospective cohort	138	Upper segment vaginal culture	33.3%	Culture-based method	Choi et al. (2023a)
Yin H et al. (2025)	China	-	177	Rectal swab	18%	Culture-based method +whole-genome sequencing	Yin et al. (2025)

Maternal colonization is not only epidemiologically relevant, but also constitutes a critical basis for early neonatal exposure and mother-to-infant vertical transmission. *E. coli* may ascend into the uterine cavity before membrane rupture, or be transmitted during delivery through direct contact between the neonate and the colonized birth canal (Wang S. et al., 2020). In one study involving 231 mothers with vaginal/rectal *E. coli* colonization, 114 neonates were found to carry strains identical to those isolated from their mothers, corresponding to a vertical transmission rate of 49.4% (Rad et al., 2018). A recent prospective study from Ethiopia reported a similar finding: among neonates born to colonized mothers, 53.9% carried strains genetically matched to the maternal isolates (Shimeles et al., 2025). Reported vertical transmission rates are summarized in Table 2.

**Table 2:** Evidence for maternal-to-infant transfer of *E. coli*.

Study (year)	Country/region	Research design	Number of infected mothers (n)	Vertical transmission rate	Detection method	Ref.
Rasa Tamelienė et al. (2012)	Lithuania	Prospective Cross-Section Study	808	21.4%	Culture-based method	Tamelienė et al. (2012)
Getnet Shimeles et al. (2025)	Ethiopia	Prospective Cross-sectional Cohort	97	57.70%	Culture-based method	Shimeles et al. (2025)
Rad et al. (2018)	Iran	Survey, descriptive analysis study	231	49.40%	Culture-based method	Rad et al. (2018)
Zhang et al. (2025)	China	Prospective cohort	201	11.0%	Culture-based method + molecular strategy	Zhang et al. (2025)
Maya Frank Wolf et al. (2021)	Israel	Prospective case–control study	34	50%	Culture-based method	Frank Wolf et al. (2021)
Koizumi et al. (2020)	Japan	Prospective cohort study	182	4.30%	Culture-based method + molecular strategy	Koizumi et al. (2020)

Although maternal colonization and vertical transmission provide the biological basis for neonatal exposure, the epidemiology of neonatal invasive *E. coli* disease needs to be considered separately because it involves different study populations, case definitions, and surveillance systems. Representative data from North America, Europe, Asia, Africa, and low- and lower-middle-income countries are summarized in Table 3. These studies collectively show that *E. coli* has become a major cause of neonatal invasive infection, particularly early-onset sepsis among preterm and very-low-birth-weight infants.

**Table 3:** Representative epidemiological evidence on neonatal invasive *E. coli* disease and invasive *E. coli* burden.

Study/source	Country/region	Study design	Population	Key findings	Refs.
Malaure et al. 2024	France/Europe	Multicenter genomic study	32 neonatal *E. coli* EOS cases	ST1193 emerged as a major clone, with meningitis and ESBL-producing isolates reported.	Malaure et al. (2024)
Stoll et al. 2020	United States/North America	Multicenter prospective surveillance	217,480 infants; 235 EOS cases	*E. coli* was the most frequent EOS pathogen, especially among preterm and VLBW infants.	Stoll et al. (2020)
Miselli et al. 2022	Italy/Europe	Prospective area-based surveillance	159,898 live births; 64 EOS cases	*E. coli* exceeded *GBS* as the leading EOS pathogen in this Italian region.	Miselli et al. (2022)
CDC Active Bacterial Core surveillance	United States/North America	Active EOS surveillance	Infants aged 0–2 days with blood/CSF isolates	*GBS* and *E. coli* are leading EOS pathogens; *E. coli* predominates in preterm and VLBW infants.	CDC (2024)
Joshi et al. 2022	United States/California	Retrospective California cohort	2,872,964 neonates; 1,535 EOS cases	EOS incidence was highest in preterm infants; *E. coli* increased over time while *GBS* decreased.	Joshi et al. (2022)
Nishihara et al. 2025	North America	Retrospective North American NICU cohort	172,921 level IV NICU infants	Infection rates increased with lower gestational age; *E. coli* increased among bloodstream isolates.	Nishihara et al. (2025)
Grome et al. 2026	United States/North America	Prospective area-based surveillance	1,345 invasive *E. coli* infections	Invasive *E. coli* incidence was 74.7/100,000; 13.8% were ESBL-producing isolates.	Grome et al. (2026)
Doenhardt et al. 2020	Germany/Europe	Single-center retrospective study	73 *E. coli* invasive infection cases within 90 days of life	*E. coli* remained clinically relevant in the post-*GBS*-screening era.	Doenhardt et al. (2020)
Lai et al. 2021	China/Asia	Retrospective neonatal invasive infection study	94 neonatal invasive *E. coli* cases	EOD and LOD had similar incidence but different risk-factor profiles.	Lai et al. (2021)
Lu et al. 2023	Taiwan, China/Asia	12-year single-center study	26 cases of early-onset neonatal sepsis caused by *E. coli*	*GBS* and *E. coli* were the main EOS pathogens; *E. coli* increased over time.	Lu et al. (2022)
Pearse et al. 2025	Malawi/Africa	Genomic epidemiological study	208 neonatal blood/CSF *E. coli* isolates	High ST and O-antigen diversity may limit O-antigen-based vaccine coverage.	Pearse et al. (2025)
Wen et al. 2021	Low- and lower-middle-income countries	Systematic review and meta-analysis	88 studies; 10,458 Gram-negative isolates	Gram-negative pathogens caused a major neonatal sepsis burden in LMICs, with high AMR.	Wen et al. (2021)

## Intrauterine infection and inflammation caused by *E. coli*

Intrauterine infection and inflammation represent one of the major mechanisms underlying infection-related adverse pregnancy outcomes (Gomez-Lopez et al., 2022; Negishi et al., 2022). The chorioamnionitis/intrauterine infection–inflammation axis plays a pivotal role in mid-trimester pregnancy loss and extremely preterm birth, and its associated pathogen spectrum is often characterized by polymicrobial infection, in which *E. coli* is one of the common causative organisms (Lukanović et al., 2023). Pathogens such as *E. coli* can ascend from the lower genital tract into the fetal membranes and amniotic cavity, triggering local and intra-amniotic inflammatory responses that may subsequently lead to the onset of uterine contractions, membrane rupture, and preterm delivery (Carter et al., 2023). In addition, previous studies have confirmed that *E. coli* is associated with a range of perinatal complications, including intrapartum fever, chorioamnionitis, miscarriage, preterm birth, and stillbirth (Shi et al., 2025). Studies of the cervical microbiota have further shown that pregnant women whose cervical secretions harbor pathogenic microorganisms-mainly *Candida* spp., *E. coli*, and *Staphylococcus aureus*-have significantly lower term delivery rates, along with increased incidences of premature rupture of membranes, intrauterine infection, and neonatal respiratory distress (Balla et al., 2024; Wang et al., 2024; Brown et al., 2019).

Overall, *E. coli* is not only a common colonizing organism in the female genital tract during pregnancy, but also an important perinatal pathogen. Its associations with preterm birth, premature rupture of membranes, and adverse maternal and neonatal infectious outcomes provide important clinical and epidemiological evidence for further investigation into its pathogenic mechanisms and virulence factors.

## Antimicrobial resistance patterns

As summarized in Table 4, neonatal invasive *E. coli* infection and EOS are consistently associated with high ampicillin resistance. Recent U. S. data still showed ampicillin resistance around 67% (Flannery et al., 2021), whereas a 20-year Spanish EOS series reported a rate of 92.8% (Mendoza-Palomar et al., 2017), indicating that resistance to first-line aminopenicillins has remained persistently high over time rather than representing an isolated finding in recent cohorts. Resistance to gentamicin and third-generation cephalosporins showed greater geographic variability. In a multicenter Chinese NICU study, cefotaxime resistance reached 46.22%, with ESBL phenotype and multidrug resistance observed in 35.68 and 36.74% of isolates, respectively (Xiao et al., 2023). Similarly, a recent Malawian genomic study reported ceftriaxone resistance in 27.8% of neonatal invasive isolates, and more than three-quarters of ceftriaxone-resistant isolates were also resistant to both ampicillin and gentamicin, supporting the view that neonatal *E. coli* resistance is evolving toward broader multidrug-resistant phenotypes (Pearse et al., 2025). As shown in Table 5, maternal colonization- and infection-associated *E. coli* isolates display a related but distinct resistance pattern. In Vietnam, maternal vaginal isolates showed high resistance to ampicillin, trimethoprim-sulfamethoxazole, gentamicin, and third-generation cephalosporins, together with an ESBL rate of 51% and an MDR rate of 56% (Viet et al., 2021). By contrast, community urine isolates from pregnant women in Burkina Faso still showed high resistance to ampicillin and cotrimoxazole, but substantially lower resistance to ciprofloxacin and gentamicin, with lower ESBL and MDR rates (Post et al., 2022). These findings suggest that the maternal reservoir of resistant *E. coli* is already established, but its magnitude varies considerably by region and clinical setting. A temporal perspective is also important for maternal isolates. An earlier study from Iraq had already identified ESBL-PCR positivity in 73.9% of vaginal *E. coli* isolates from pregnant women (Al-Mayahie, 2013a), whereas a more recent study from Somalia documented high resistance to ceftriaxone, ceftazidime, ciprofloxacin, and sulfamethoxazole in symptomatic maternal urinary isolates (Mohamed et al., 2023). Taken together, the resistance burden in perinatal *E. coli* is not restricted to neonatal invasive disease alone, but extends across the maternal reservoir and, over time, increasingly includes third-generation cephalosporin resistance, ESBL production, and multidrug resistance.

**Table 4:** Antimicrobial resistance profiles reported in studies of neonatal/perinatal *Escherichia coli* infection.

Region/study design	Sample size/population	Ampicillin resistance	Gentamicin resistance	Third-generation cephalosporin resistance	Carbapenem resistance	Other key findings	Study period	Ref
United States/multicenter prospective EOS surveillance	86 *E. coli* EOS cases	74.1%; 83.1% in preterm infants; 37.5% in term infants	Approximately 10.0%	Approximately 4.7%	–	Dual resistance to ampicillin plus gentamicin was 7.8%; Among infants whose mothers received intrapartum ampicillin, 81.8% of isolates were ampicillin-resistant	2015–2017	Stoll et al. (2020)
United States/single-center 15-year retrospective cohort	68 neonatal *E. coli* bloodstream/CSF infections; 38 EOS and 30 LOS cases	Overall 70.0%; EOS 74.0%; LOS 66.0%	Overall approximately 7.5%; EOS 8.0%; LOS 7.0%	–	–	No significant change in resistance rates over the 15-year period; Ampicillin resistance was not significantly associated with mortality or severe illness	2008–2022	Hoffman et al. (2024)
Spain/single-center 20-year EOS trend study	78 *E. coli* EOS cases	75.4% (2010–2014: 92.8%)	12.3% (2010–2014: 28.6%)	1.5%	0%	Incidence and mortality were highest in very-low-birth-weight infants	1994–2014	Mendoza-Palomar et al. (2017)
China/multicenter NICU isolate study	370 neonatal *E. coli* isolates from blood, CSF, sputum, gastric fluid, and other secretions	–	29.73%	46.22% (cefotaxime)	1.35%	MDR 36.74%; ESBL phenotype 35.68%	2015–2020	Xiao et al. (2023)
South Asia/systematic review	2,798 *E. coli* isolates	88.20%	67.90%	66.9% (cefotaxime)	8.1% (meropenem)	MDR 54.0%	Approximately 2000–2018	Chaurasia et al. (2019)
Germany/single-center neonatal sepsis surveillance	23 *E. coli* isolates	74.00%	0%	8.7% (cefuroxime)	0% (imipenem)	No resistance to gentamicin or imipenem was detected	2012–2020	Tessema et al. (2021)
Malawi/genomic study of invasive neonatal *E. coli* disease	Phenotypic testing numbers varied by drug: ampicillin n = 190, gentamicin n = 197, ceftriaxone n = 198; WGS n = 169	75.3%	24.9%	27.8% (ceftriaxone)	0% (meropenem)	Among ceftriaxone-resistant isolates, 76.4% were also resistant to both ampicillin and gentamicin	2012–2021	Pearse et al. (2025)
Ethiopia/cross-sectional study of suspected neonatal sepsis	19 *E. coli* isolates	68%	58%	100% (ceftazidime); 68% (cefotaxime)	–	–	2020	Sherif et al. (2023)
United States/multicenter EOS surveillance	235 EOS cases; antimicrobial susceptibility data available for approximately 81 *E. coli* isolates	77.8%	10.0%	5.4% (ceftriaxone, 3/56)	0% (carbapenems)	Dual non-susceptibility to ampicillin plus gentamicin was 8.9% (7/79); All 3 ESBL-producing isolates were *E. coli*	2015–2017	Flannery et al. (2022b)
United States/single-center neonatal bacteremia study	43 neonatal *E. coli* bacteremia cases	67.0%	14.0%	2.0% (ceftriaxone)	–	One ESBL-producing isolate was identified; mortality was 28%, and all deaths occurred in preterm infants	2006–2016	Cole et al. (2019)
Türkiye/single-center 7-year EOS study	101 culture-proven EOS cases; 29 *E. coli* isolates	72.4%	6.9%	–	–	ESBL-producing *E. coli* accounted for 27.6%	2013–2020	Topcuoglu et al. (2022)
Ethiopia/cross-sectional study of suspected neonatal bloodstream infection	18 *E. coli* isolates	66.7%	61.1%	44.4% (ceftriaxone)	27.8% (meropenem)	MDR 61.1%	2019–2021	Deress et al. (2024)
Ethiopia/retrospective study from two NICUs	19 *E. coli* isolates	–	33.0%	44.0% (ceftriaxone) 25.0%(ceftazidime)	19.0% (meropenem)	Ertapenem resistance 7.0%; amikacin resistance 6%; MDR approximately 32%	2023	Mamo et al. (2025)

**Table 5:** Antimicrobial characteristics of maternal *E. coli* colonization/infection in pregnancy-related studies.

Region/clinical setting	*E. coli*-related samples	Main antimicrobial findings	ESBL/MDR	Ref
Ethiopia/late-pregnancy vaginal colonization	*E. coli* 82/274 (29.9%)	Universal susceptibility to gentamicin, ceftriaxone, and imipenem; low resistance to ciprofloxacin and cephalosporins; cotrimoxazole resistance 28.1%	-	Birhane Fiseha et al. (2021)
Taiwan, China/rectovaginal colonization screening during pregnancy	*E. coli* detected in 62/137; pathogenic *E. coli* in 49/137	Lower susceptibility in pathogenic isolates to ampicillin/sulbactam, piperacillin, and TMP-SMX; all isolates remained susceptible to cefepime, carbapenems, and amikacin	-	Liu et al. (2019)
Spain/vaginal colonization versus obstetric infection isolates	82 vaginal isolates and 63 obstetric infection isolates	Overall ampicillin resistance was 65%; gentamicin resistance was higher in obstetric infection isolates than in vaginal colonization isolates	-	Sáez-López et al. (2016b)
Vietnam/hospitalized pregnant women with vaginal swabs	*E. coli* 667/3,104 (21%)	High resistance to ampicillin (92%), 3GCs (~56%), gentamicin, TMP-SMX, and ciprofloxacin; carbapenem resistance	ESBL 51%; MDR 56%	Viet et al. (2021)
Burkina Faso/community urine cultures from pregnant women	155 *E. coli* isolates	Resistance rates were 65.8% to ampicillin, 64.4% to cotrimoxazole, 16.2% to ciprofloxacin, and 3.9% to gentamicin	ESBL 3.2%; MDR 5.2%	Post et al. (2022)
Iraq/symptomatic high vaginal swabs	19 isolates	Resistance rates were 100% to cefotaxime, 57.8% to ceftazidime, 42.1% to ceftriaxone, 36.8% to gentamicin, 31.5% to ciprofloxacin, 31.5% to TMP-SMX, and 100% to amoxicillin-clavulanate; no resistance to imipenem or meropenem was detected	ESBL-PCR positivity 73.9%; MDR 36.8%	Al-Mayahie (2013a)
Somalia/urinary tract infection in pregnancy	*E. coli* 42/220 (19.0%)	Resistance rates were 92.9% to ampicillin, 78.2% to ceftriaxone, 76.2% to ceftazidime, 64.3% to ciprofloxacin, 90.5% to sulfamethoxazole, 23.8% to gentamicin, 16.7% to meropenem, and 16.7% to amoxicillin/clavulanate	-	Mohamed et al. (2023)
Adigrat, Ethiopia; asymptomatic bacteriuria	19 *E. coli* isolates	Ampicillin resistance 100%; moderate resistance to TMP-SMX (47.4%), gentamicin (21.1%), and ciprofloxacin (21.5%); low ceftriaxone resistance (5.2%)	-	Tadesse et al. (2018)
Ethiopia/asymptomatic bacteriuria	22 *E. coli* isolates	Resistance rates were 86.4% to amoxicillin and 77.7% to cotrimoxazole; susceptibility to gentamicin and nitrofurantoin was 91.0%	MDR 57.1%	Wabe et al. (2020)
Ethiopia/asymptomatic bacteriuria	17 *E. coli* isolates	Resistance rates were 100% to ampicillin, 70.6% to cephalothin, 76.4% to amoxicillin-clavulanate, 53.0% to sulfisoxazole, 17.6% to ceftriaxone, 11.8% to gentamicin, and 5.9% to ciprofloxacin	MDR 88.2%	Bizuwork et al. (2021)
Spain/severe obstetric infections	78 *E. coli* isolates	Resistance rates were 63% to ampicillin, 13% to amoxicillin-clavulanate, 22% to cefazolin, 3% to cefotaxime, 3% to ciprofloxacin, 4% to gentamicin, 28% to TMP-SMX, and 0% to imipenem	MDR 26%	Guiral et al. (2015)

## Neonatal sepsis caused by *E. coli*

*E. coli* is one of the major Gram-negative pathogens causing neonatal sepsis, particularly EOS (Stoll et al., 2020; Miselli et al., 2022), although the epidemiology varies substantially by region and setting, evidence from developing countries suggests that *E. coli* is among the three leading neonatal sepsis pathogens, after *Klebsiella pneumoniae* and *Staphylococcus aureus*, accounting for roughly 15% of cases overall (Wen et al., 2021; Waters et al., 2011; Zelellw et al., 2021). Maternal vaginal/rectal colonization and subsequent perinatal vertical transmission constitute key mechanisms underlying neonatal *E. coli* infection (Fleiss et al., 2023). *E. coli*-associated chorioamnionitis may lead to fetal bacteremia before delivery, and perinatal maternal bacteremia is also closely associated with EOS; neonatal bloodstream infection often presents on the day of birth (Fleiss et al., 2023; Gad et al., 2024). In addition, *E. coli* colonizing the vagina or rectum of asymptomatic pregnant women may be transmitted to the neonate during delivery, resulting in EOS manifesting as sepsis or meningitis and significantly increasing the risk of neonatal blood or cerebrospinal fluid infection (Malaure et al., 2024; Liu et al., 2019). The risk of *E. coli* infection is further increased in preterm infants, very low birth weight infants, and extremely preterm infants (Puopolo et al., 2018). As an important member of extraintestinal pathogenic ExPEC, *E. coli* possesses substantial virulence and resistance potential and represents a major pathogenic group in neonatal sepsis (Dale and Woodford, 2015). In some neonatal cohorts, the rate of death or severe adverse outcomes among infants with *E. coli* sepsis has exceeded 40%, with the risk increasing significantly as birth weight decreases (Stoll et al., 2020; Hoffman et al., 2024). Clinically, neonatal *E. coli* infection may present fulminantly and can progress early to septic shock; in preterm infants, it is often accompanied by acute respiratory decompensation requiring ventilatory support, and may be complicated by thrombocytopenia and disseminated intravascular coagulation (Hoffman et al., 2024; Simonsen et al., 2014; Oldendorff et al., 2022). Survivors may also suffer long-term neurological sequelae. Although term infants generally have a better overall prognosis, those complicated by meningitis remain at risk for long-term outcomes such as hearing loss and developmental delay. These findings underscore that even in the era of *GBS* prophylaxis, and in the absence of obvious intrapartum risk factors, clinicians should maintain a high index of suspicion for *E. coli* infection in neonates who develop signs of infection within the first few days after birth.

## Neonatal meningitis caused by *E. coli*

*E coli* is a major causative pathogen of neonatal purulent meningitis. It is the second most common pathogen in term infants and the leading pathogen in preterm infants; together with *GBS*, it accounts for approximately 60% of all cases. The overall mortality rate of this disease is about 10–15%, and 30–50% of survivors may be left with severe neurological sequelae, including hearing loss, impaired motor function, and developmental disorders (Nhu et al., 2024). Studies of maternal vaginal/rectal isolates suggest that some colonizing strains harbor virulence determinants associated with invasive neonatal disease and may be vertically transmitted during perinatal exposure; these virulence factors are discussed in detail in the later section “Pathogenesis and Virulence Gene Profile of Perinatal *E. coli* Infection” (Kaczmarek et al., 2018). Surveillance data from eastern China indicate that *E. coli* remains an important cause of neonatal meningitis (Liu et al., 2021). In a Canadian multicenter cohort of infants younger than 90 days with proven or suspected meningitis, *E. coli* and *GBS* were the two most common pathogens overall; however, because of the limited sample size and mixed case definition, these findings should be interpreted cautiously (Ouchenir et al., 2017).

In this context, early- and late-onset neonatal meningitis should be clearly distinguished based on the timing of disease onset. In many neonatal infection studies, early-onset disease is defined as onset within the first 72 h after birth, whereas late-onset disease is defined as onset after 72 h of life (Korang et al., 2021). Notably, the relative contributions of *GBS* and *E. coli* to early- and late-onset meningitis appear to vary across different settings. French national surveillance data revealed *GBS* as the predominant pathogen in both early (0–4 days after birth) and late (5–28 days after birth) onset neonatal meningitis, although *E. coli* was more frequently observed among preterm infants (Gaschignard et al., 2011). In contrast, a multicenter study from Shanghai reported *GBS* as the leading pathogen in early-onset (0–4 days after birth) meningitis, while *E. coli* was the predominant pathogen in late-onset disease (Xu et al., 2019). Collectively, these findings indicate that *E. coli* represents an important pathogen in neonatal meningitis, particularly in preterm infants and in certain late-onset cohorts; however, its predominance over *GBS* is not universal across all settings.

Neonatal bacterial meningitis caused by *E. coli* often has an insidious onset and lacks specific clinical manifestations, frequently presenting with sepsis-like symptoms rather than classic meningeal signs. Common manifestations include fever or hypothermia, feeding difficulties, irritability or lethargy, somnolence, and seizures (van der Flier, 2021). In contrast, typical signs of meningitis, such as bulging fontanelle and neck stiffness, are often absent or only mildly evident in neonates (van der Flier, 2021; Mathias et al., 2024). Even with timely, adequate, and appropriate antibiotic therapy, a substantial proportion of affected infants (>30%) still progress to structural complications such as ventriculitis, brain abscess, and hydrocephalus. Accordingly, *E. coli* meningitis is widely recognized as a severe infectious disease with a high propensity to cause neonatal brain injury (Basmaci et al., 2015; Asgedom et al., 2025; García et al., 2024; Hsu et al., 2018). A French study reported an overall mortality rate of 9.2%, with a significantly higher risk of death among preterm infants. The rate of acute complications was 33.2%, most commonly seizures, intracranial hemorrhage, and hydrocephalus, while infants with persistently positive cerebrospinal fluid cultures were more likely to develop acute complications and permanent neurological sequelae (Basmaci et al., 2015). In addition, compared with matched controls without meningitis, survivors of bacterial meningitis in infancy had a significantly increased long-term mortality risk, as well as higher rates of neurodevelopmental abnormalities such as cerebral palsy, intellectual disability, and sensory impairment (Snoek et al., 2022).

Population-level studies of invasive *E. coli* strains have confirmed that the K1 capsule is a core virulence determinant closely associated with neonatal bacteremia and meningitis. *In vitro* functional studies have shown that K1 capsule synthesis can markedly enhance the survival of *E. coli* in human serum; its polysialic acid structure inhibits complement activation and phagocytosis, thereby enabling the organism to evade host defenses effectively in the bloodstream and central nervous system (Arredondo-Alonso et al., 2023). Available human data specifically quantifying CSF bacterial burden in *E. coli* meningitis are limited; an often-cited pediatric study reported that CSF bacterial counts in bacterial meningitis ranged up to 4 × 10^9 CFU/mL, and cases caused by *E. coli* K1 had counts of at least 10^7 CFU/mL (Bingen et al., 1990). A study including four cases of recurrent neonatal *E. coli* meningitis found that all infants relapsed within 2–28 days after completing an approximately 3-week standard antibiotic course. The isolates recovered at recurrence had antimicrobial susceptibility profiles identical to those of the initial isolates, and in one case, whole-genome sequencing confirmed that both episodes were caused by the same strain (Vissing et al., 2021). Notably, *E. coli* remains the most common pathogen responsible for recurrent neonatal meningitis. Previous studies have shown that even after appropriate antimicrobial treatment, the recurrence rate may still reach approximately 6–21% (Vissing et al., 2021; Snoek et al., 2023).

## Pathogenesis and virulence gene profile of perinatal *E. coli* infection

*E. coli* strains associated with perinatal infection predominantly belong to the category of ExPEC, and their pathogenicity largely depends on the combination and coordinated expression of specific virulence genes (Sáez-López et al., 2016b; Bonacorsi and Bingen, 2005; Vila et al., 2016). Among ExPEC, the lineages most relevant to maternal and neonatal infections are UPEC and neonatal NMEC (Dale and Woodford, 2015; Vila et al., 2016). Current evidence suggests that vaginal *E. coli* isolates from pregnant women often display ExPEC-like virulence gene profiles, indicating that they are not merely commensals but potential reservoirs of strains capable of causing obstetric and neonatal infection (Sáez-López et al., 2016b; Sáez-López et al., 2016a). Functionally, ExPEC virulence determinants can be grouped into factors mediating adhesion and colonization, immune evasion/protection, iron acquisition, toxin production and invasion; in addition, many of these traits are carried on pathogenicity-associated genomic islands and are enriched within internationally disseminated high-risk clonal backgrounds (Sora et al., 2021; Vila et al., 2016; Desvaux et al., 2020; Kocsis et al., 2022).

## Virulence genes related to adhesion and colonization

Adhesion is a key early step in ExPEC pathogenesis and likely contributes to vaginal colonization and subsequent ascending obstetric and neonatal infection. Vaginal *E. coli* isolates obtained during pregnancy often carry ExPEC-associated virulence profiles, including adhesins such as FimH and PapG, protectins such as Iss and TraT, and toxins such as Usp and Vat, supporting their pathogenic potential (Sora et al., 2021; Boutouchent et al., 2024; Sáez-López et al., 2016b; Al-Mayahie, 2013b). The important adhesion-related genes include fimH, which encodes the type 1 fimbrial adhesin; the pap gene cluster associated with P fimbriae; the sfa gene associated with S fimbriae; and other adhesin genes such as afa/dr and iha. These adhesive structures are largely assembled through the chaperone–usher pathway and constitute a key early molecular basis for the initiation of ExPEC infection (Sora et al., 2021). Among them, FimH is one of the best-characterized adhesins. It not only contributes to extraintestinal colonization, but also facilitates the persistence of ExPEC within the competitive intestinal environment. In UPEC, FimH can further specifically recognize mannosylated receptors on the urothelial surface, including uroplakin Ia (UPIa), thereby promoting bacterial adhesion, internalization, and activation of inflammatory responses (Silmon de Monerri et al., 2025; Russell et al., 2019; Russell et al., 2018). Recent evidence also links FimH to ascending urinary tract infection and kidney colonization. Tseng et al. showed that fimH and papGII co-carriage was associated with upper urinary tract infection and that FimH acted synergistically with PapGII to promote the establishment and maintenance of *E. coli* kidney infection. Thus, FimH-mediated adhesion may support progression from lower urinary tract colonization to upper urinary tract infection, a potential prerequisite for invasive disease (Tseng et al., 2022). Perinatal studies have likewise shown that vaginal *E. coli* isolates are commonly enriched in such adhesion-associated genes. Metagenomic and isolate-based studies have found that the carriage rate of fimH in vaginal isolates from pregnant women may reach approximately 90%, while pap-related genes are also frequently detected in pregnancy-associated isolates and, in some studies, are more prevalent than in non-pregnant controls (Boutouchent et al., 2024; Al-Mayahie, 2013b; Guiral et al., 2011; Watt et al., 2003). These findings suggest that adhesion-related virulence genes may confer a stronger capacity for persistent vaginal colonization, thereby creating favorable conditions for subsequent chorioamniotic invasion and neonatal epithelial colonization.

## Genes related to immune evasion and protection

After colonization, *E. coli* must overcome complement-mediated killing, phagocytosis, and other innate immune defenses to persist in the bloodstream and establish invasive extraintestinal infection (Abreu and Barbosa, 2017; Biran and Ron, 2018). Among the most important protective determinants are capsule-associated genes, particularly the K1 capsule locus (kps genes and related biosynthetic determinants such as neu), because recent evolutionary and functional analyses confirmed that K1 capsule expression is strongly linked to invasive lineages and directly enhances survival in human serum (Arredondo-Alonso et al., 2023). Consistent with this, ExPEC can upregulate extracytoplasmic polysaccharide biosynthesis in response to bloodstream-associated signals, thereby increasing serum resistance and persistence during bacteremia (Ma et al., 2018). Another key immune-protective determinant is iss (increased serum survival), which contributes to capsule production and protects bacteria from the bactericidal activity of complement (Biran et al., 2021). More recently, nhaA has also been identified as a contributor to immune evasion, as it helps maintain envelope integrity and reduces susceptibility to complement attack, thereby promoting ExPEC virulence (Mao et al., 2023). In NMEC, additional factors such as ompA and ibeA further enhance pathogenic fitness by promoting interaction with human brain microvascular endothelial cells and facilitating traversal of the blood–brain barrier (Maruvada and Kim, 2012). Collectively, these genes form a coordinated protection network that enables pathogenic *E. coli* to progress from mucosal colonization to bloodstream dissemination and, in some cases, central nervous system invasion.

## Iron acquisition-related virulence genes

Iron acquisition systems represent one of the most stable and most prevalent virulence-associated modules in ExPEC. Comparative pathogenomic studies have shown that pathogenic *E. coli*, particularly ExPEC, are significantly more enriched in iron uptake-related genes than commensal strains, and that most ExPEC isolates simultaneously harbor multiple iron acquisition loci, underscoring the recurrent importance of this module in the ExPEC virulence repertoire (Clark and Maresso, 2021; Galardini et al., 2020). Because free iron is highly restricted within the host, pathogens must overcome so-called nutritional immunity through efficient iron scavenging systems in order to maintain growth and establish infection *in vivo* (Murdoch and Skaar, 2022; Robinson et al., 2018). In perinatal- and neonatal-associated *E. coli*, iron acquisition genes are among the most frequently detected virulence gene categories. In a metagenomic study of vaginal *E. coli* during pregnancy, fyuA, iutA, and fepA were identified as major iron uptake-related genes, with detection rates of 91 and 88% for fyuA and fepA, respectively (Boutouchent et al., 2024). Bacterial iron acquisition systems facilitate colonization and biofilm formation by sequestering iron from host-associated proteins (Kang and Kirienko, 2018; Oliveira et al., 2021; Chan et al., 2023). Previous studies have further reported detection rates of approximately 42, 40, and 38% for iucC, fyuA, and iutA (Sáez-López et al., 2016a), respectively, whereas in other cohorts the carriage rate of fyuA approached 80% (Sáez-López et al., 2016b). Host iron overload and impaired immune function may further increase neonatal susceptibility to *E. coli* K1 sepsis (Michels et al., 2019). Collectively, these findings suggest that even when certain vaginal colonizing strains carry fewer virulence genes overall than classical invasive strains, they may still retain the most critical metabolic virulence advantage of ExPEC, namely the ability to compete for and acquire iron under host-restricted conditions. In NMEC, iron-limited conditions may further regulate invasion-associated phenotypes, thereby enhancing the ability of the organism to penetrate the blood–brain barrier (Zheng et al., 2024).

## Toxin- and invasion-associated virulence genes

Toxin- and invasion-associated virulence genes are major determinants of the capacity of ExPEC to damage host tissues and disseminate across biological barriers. The best-characterized toxin genes include hlyA and cnf1, encoding *α*-hemolysin (HlyA) and cytotoxic necrotizing factor 1 (CNF1), respectively. HlyA is a pore-forming toxin that disrupts host cell membranes, induces Ca^2+^ influx, and perturbs intracellular signaling pathways, thereby triggering pronounced inflammation and tissue injury (Watt et al., 2003; Massella et al., 2021; Schulz et al., 2021; Dhakal and Mulvey, 2012; Wang C. et al., 2020). CNF1 promotes bacterial invasion by modulating Rho GTPase signaling and remodeling the host actin cytoskeleton, which facilitates epithelial barrier penetration (Carlini et al., 2021; Tsoumtsa Meda et al., 2022). These two toxins frequently coexist in highly virulent UPEC and other invasive ExPEC isolates and are commonly considered a characteristic “dual-toxin” profile linked to increased pathogenicity (Clark and Maresso, 2021; Garcia et al., 2013). In perinatal settings, hlyA and cnf1 have also been detected in vaginal *E. coli* isolates, although their prevalence varies among studies; reported rates range from approximately 21% for hlyA in some cohorts to 40–45% for hlyA and/or cnf1 in isolates from pregnant women in others (Sáez-López et al., 2016a; Hilbert et al., 2008). Moreover, both hlyA and cnf1 are more prevalent in ExPEC than in commensal intestinal isolates and are significantly enriched in bloodstream isolates, further supporting their contribution to neonatal sepsis and meningitis (Nagarjuna et al., 2015; Bhattacharjee et al., 2023; Bok et al., 2020). Among invasion-associated factors, ibeA is of particular importance. Among invasion-associated factors, ibeA is of particular importance. The ibeA-encoded invasion of brain endothelium protein A promotes bacterial invasion of brain microvascular endothelial cells and is considered to contribute to traversal of the blood–brain barrier (Huang et al., 2001; Huang et al., 2000). In addition, *E. coli* infection of human brain microvascular endothelial cells activates Egr-1, a key regulator of blood–brain barrier integrity, which increases barrier permeability and amplifies neuroinflammatory signaling by targeting VEGFA, PDGFB, and ANGPTL4 (Yang et al., 2024).

## Pathogenicity Islands, phylogenetic background, and high-risk clones

Beyond individual virulence genes, the genomic organization and clustering of virulence determinants are also critical to ExPEC pathogenicity. Many ExPEC-associated virulence factors are located on pathogenicity islands (PAIs) and other mobile genetic elements, which can be acquired through horizontal gene transfer and enable a single strain to simultaneously gain multiple pathogenic traits, including adhesins, capsules, toxins, and iron acquisition systems (Desvaux et al., 2020; Messerer et al., 2017; Biggel et al., 2020; da Silva et al., 2017). Several studies have shown that vaginal *E. coli* isolates from pregnant women carry more PAI-associated genes than isolates from non-pregnant women, particularly genes enriched in regions encoding fimbriae, hemolysin, and CNF1 (Sáez-López et al., 2016b; Guiral et al., 2011; Rashki, 2014). This finding suggests that highly virulent lineages may be preferentially adapted to, or selectively favored within, the vaginal microenvironment during pregnancy (Williams et al., 2021; Boutouchent et al., 2024). From a phylogenetic perspective, perinatal-associated *E. coli* isolates are more commonly assigned to the B2 and D phylogroups, which are strongly associated with ExPEC virulence, rather than to the generally less virulent commensal-associated A and B1 groups. In several studies, the proportion of phylogroup B2 among vaginal *E. coli* colonizing (VEC) isolates reached 52% or higher, and metagenomic analyses have likewise demonstrated a clear predominance of B2 and D phylogroups in vaginal isolates from pregnant women (Boutouchent et al., 2024; Sáez-López et al., 2016b). In addition, sequence types such as ST95, ST131, and ST1193 belong to globally disseminated high-risk ExPEC lineages. Among these, ST95 has long been linked to NMEC, whereas ST1193 has emerged rapidly in recent years as a successful B2-associated epidemic clone and has been reported in early-onset neonatal sepsis, often in association with an ESBL phenotype (Nhu et al., 2024; Pitout et al., 2022; Xia et al., 2022). Collectively, these findings indicate that the pathogenic potential of perinatal-associated *E. coli* depends not only on individual virulence genes, but also on its broader phylogenetic background and high-risk clonal structure.

## Host–pathogen interactions

Host–pathogen interactions are central to determining whether maternal *E. coli* colonization remains asymptomatic or progresses to invasive neonatal disease. At the maternal–fetal interface, pregnancy requires a finely regulated immune balance that preserves fetal tolerance while maintaining antimicrobial defense (Alippe et al., 2025; Megli and Coyne, 2022). More broadly, the importance of vaginal colonization as a precursor to ascending intrauterine infection has been recognized across perinatal bacterial infections (Brokaw et al., 2021). In this setting, maternal gastrointestinal and genitourinary colonization provides the initial reservoir for perinatal exposure, while disruption of the vaginal microenvironment may facilitate persistence of virulent strains (Boutouchent et al., 2024; Hudson et al., 2020). Adhesion-associated factors such as type 1 fimbriae, P fimbriae, and S fimbriae further support sustained mucosal colonization and create favorable conditions for ascending infection and neonatal exposure during delivery (Sora et al., 2021; Boutouchent et al., 2024; Russell et al., 2018). Once *E. coli* ascends into the uterine cavity or reaches the fetal membranes, host inflammatory responses become a major determinant of pathological progression. Bacterial components, particularly lipopolysaccharide, activate innate immune pathways in the cervix, decidua, and fetal membranes, resulting in cytokine release, leukocyte infiltration, and chorioamnionitis (Cappelletti et al., 2020; Olmos-Ortiz et al., 2022). This inflammatory process contributes to membrane weakening, uterine activation, and preterm birth, underscoring that adverse pregnancy outcomes are driven not only by bacterial presence itself but also by dysregulated host responses at the maternal–fetal interface (Carter et al., 2023; Shi et al., 2025). Experimental and translational studies further suggest that once bacteria enter the amniotic cavity, intra-amniotic inflammation may contribute not only to acute fetal injury but also to later developmental abnormalities (Motomura et al., 2021; Bhagirath et al., 2021; Šket et al., 2021). Following vertical transmission, progression from exposure to invasive neonatal infection depends largely on the balance between bacterial fitness and neonatal immune defense (Shimeles et al., 2025; Wang S. et al., 2020). This is particularly relevant in preterm and very-low-birth-weight infants, whose early host defense is compromised by attenuated phagocyte responses and impaired inflammatory signaling (Wisgrill et al., 2016; Prosser et al., 2013). Maternal immune factors, including transferred antibodies, contribute to neonatal protection against bacterial infections (Sereme et al., 2024b). In experimental models, maternally induced antibodies can also protect newborns against severe *E. coli* K1 infection (Sereme et al., 2024a). Under these conditions, virulence determinants involved in immune evasion and bloodstream survival become especially important. Capsule-associated traits, particularly the K1 capsule, enhance resistance to complement-mediated killing and phagocytosis, while iron acquisition systems help sustain bacterial growth under host-imposed nutritional immunity (Arredondo-Alonso et al., 2023; Clark and Maresso, 2021; Zheng et al., 2024).

Emerging evidence further suggests that host metabolic status may influence the severity of NMEC infection. Low blood leucine levels have been shown to enhance NMEC virulence through the Lrp–NsrP–purD regulatory axis, thereby promoting metabolic adaptation, bacterial survival, and replication in the bloodstream (Sun et al., 2025).

Central nervous system invasion represents a further stage in this host–pathogen interaction continuum. In NMEC, factors such as *OmpA* and *IbeA* promote adhesion to and invasion of brain microvascular endothelial cells, thereby facilitating traversal of the blood–brain barrier (Maruvada and Kim, 2012; Cieza et al., 2015). At the same time, inadequate early immune control in susceptible neonates may permit rapid bacterial proliferation in blood and subsequent dissemination to the meninges (Villavicencio-Carrisoza et al., 2025). Clinically, neonatal *E. coli* central nervous system infection can progress rapidly and may be associated with severe complications or recurrence (Saleh et al., 2024).

**Figure 1:**
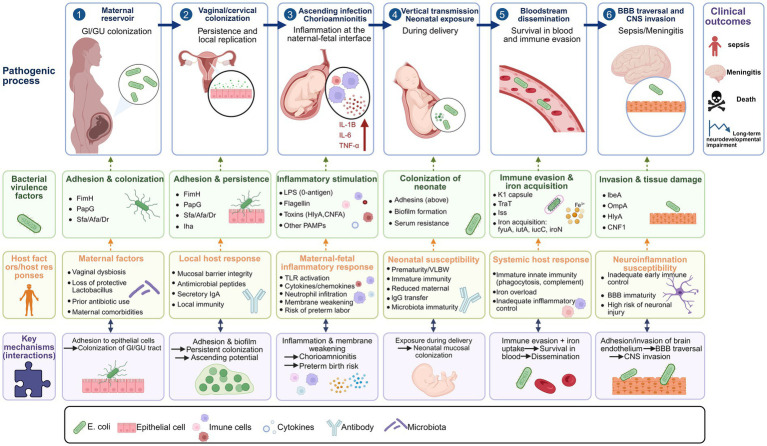
Stepwise pathogenesis of perinatal *Escherichia coli* infection and host-pathogen.

The pathogenic outcome of perinatal *E. coli* infection is also shaped by the broader genomic background of the infecting strain. Certain clonal lineages, particularly ST95 and the emerging ST1193, appear especially well adapted as neonatal pathogens (Pitout et al., 2022; Xia et al., 2022). ST95, which belongs to phylogroup B2, has long been associated with NMEC and carries a broad repertoire of virulence determinants, including the K1 capsule (Xia et al., 2022). By contrast, ST1193 represents an emerging high-risk ExPEC clone whose success appears to reflect the coupled evolution of phylogenetic background, virulence plasmids, and resistance structure (Wyrsch et al., 2022). Other high-risk clonal backgrounds, including ST131, have also been implicated in neonatal meningitis, suggesting that invasive neonatal *E. coli* is not confined to a single lineage (De Francesco et al., 2023). Accordingly, severe perinatal *E. coli* infection should be understood not as an isolated neonatal event, but as the result of sequential interactions among maternal reservoirs, bacterial virulence, inflammatory activation at the maternal–fetal interface, and immature neonatal host defense (Figure 1).

## Prevention-oriented targets and investigational strategies

Although no *E. coli*-specific preventive strategy is currently established for routine perinatal use, several investigational approaches have begun to emerge. Ecological or microbiota-based intervention is one potential direction because maternal intestinal and genital reservoirs represent important sources of neonatal exposure. In an *in vitro* intestinal microbiota model derived from pregnant donors, a phage targeting NMEC K1 reduced the bacterial burden, although the effect was incomplete and donor-dependent (Antoine et al., 2023). This finding suggests that phage-based ecological intervention may be a potential strategy for reducing maternal carriage of high-risk neonatal *E. coli* strains, but its safety, durability, and clinical feasibility during pregnancy remain to be established. Anti-adhesion strategies also represent an important prevention-oriented approach. Because FimH-mediated adhesion contributes to intestinal and urinary tract colonization and may facilitate ascending infection, FimH antagonists have been explored as non-antibiotic interventions. A high-affinity mannoside FimH antagonist was shown to selectively deplete uropathogenic *E. coli* from the gut while treating urinary tract infection in experimental models, with limited disruption of the gut microbiota (Spaulding et al., 2017). In addition, a first-in-human phase 1 study of an adjuvanted *E. coli* adhesin vaccine reported acceptable safety and immunogenicity in healthy women with or without histories of recurrent urinary tract infection (Eldridge et al., 2021). However, these anti-adhesion approaches have mainly been evaluated in urinary tract infection settings and have not yet been validated for preventing maternal genital colonization, vertical transmission, or neonatal invasive disease.

antigens should also be regarded not merely as theoretical targets but as clinically evaluated vaccine antigens for ExPEC. A tetravalent O-antigen bioconjugate vaccine, ExPEC4V, containing O1A, O2, O6A, and O25B antigens, showed acceptable safety and immunogenicity in a phase 1b trial among women with recurrent urinary tract infection and in a phase 2 randomized trial among healthy adults (Huttner et al., 2017; Frenck et al., 2019). Additional phase 1 data in healthy Japanese adults further supported the tolerability and immunogenicity of ExPEC4V (Inoue et al., 2018). More recently, the 9-valent O-antigen-based ExPEC9V candidate was evaluated in a placebo-controlled phase 3 study assessing immunogenicity and safety when co-administered with high-dose influenza vaccine in older adults, indicating that O-antigen-based ExPEC vaccination has advanced into late-stage clinical development (Leroux-Roels et al., 2026). Nevertheless, these vaccine studies were conducted mainly in adult or older-adult populations and were not designed to assess prevention of maternal colonization, vertical transmission, or neonatal invasive *E. coli* disease.

Another emerging concept is maternal immunity. Recent evidence suggests that vertically transferred maternal anti-*E. coli* IgG contributes to neonatal protection, and that reduced anti-*E. coli* antibody levels and impaired opsonization activity may be associated with neonatal *E. coli* sepsis (Diep et al., 2026). This finding supports the broader rationale for maternal-immunity-based prevention strategies, but whether vaccination or microbiota-directed approaches can safely and effectively enhance protective anti-*E. coli* immunity during pregnancy remains unknown. Therefore, phage-based ecological intervention, anti-adhesion strategies, O-antigen-based vaccines, and maternal-immunity-based approaches provide promising prevention-oriented directions, but their application to perinatal and neonatal *E. coli* infection remains investigational and requires further validation in pregnancy- and neonate-relevant models.

## Limitations

This review has several limitations. First, most of the available evidence derives from retrospective or observational studies, and substantial heterogeneity exists across studies in terms of sample sources, detection methods, strain typing approaches, and outcome definitions, which limits direct comparison of findings. Second, the current evidence base is drawn largely from selected countries and single-center cohorts, resulting in limited geographic representativeness and restricting the generalizability of the conclusions. Third, current understanding of E.coli virulence mechanisms and maternal–neonatal transmission still relies heavily on *in vitro* experiments, animal models, and genomic analyses, whereas clinically translatable evidence remains relatively limited. Finally, high-quality prospective studies and unified evidence-based guidelines are still lacking for screening strategies, optimization of empirical antimicrobial therapy, and preventive interventions in perinatal *E. coli* infection. Accordingly, many of the conclusions summarized here require further validation in future multicenter studies with larger sample sizes.

## Conclusion

Perinatal *E. coli* infection should be understood as a stepwise pathogenic continuum rather than as a single neonatal event. Maternal gastrointestinal and genital tract colonization, persistence of virulent ExPEC lineages, inflammatory activation at the maternal-fetal interface, vertical transmission, and the limited immune capacity of preterm neonates together create the conditions for progression from asymptomatic exposure to neonatal sepsis and meningitis. This integrated framework helps explain why only a subset of colonized mother-infant pairs develop invasive disease and why the most severe outcomes are concentrated in preterm and very-low-birth-weight infants. At the same time, increasing antimicrobial resistance, particularly ESBL production and multidrug resistance, has further complicated empirical therapy and heightened the clinical burden of perinatal *E. coli* infection. Therefore, future research should move beyond isolated descriptions of colonization, virulence genes, or neonatal outcomes, and instead integrate maternal reservoir characteristics, vertical transmission dynamics, bacterial pathogenic profiles, neonatal susceptibility, and regional resistance patterns into a unified risk model. Such an approach will be essential for improving early risk stratification, prevention strategies, and targeted treatment in both maternal and neonatal care.

## References

[ref1] Abreu A. G. Barbosa A. S. (2017). How *Escherichia coli* circumvent complement-mediated killing. Front. Immunol. 8:452. doi: 10.3389/fimmu.2017.00452, 28473832 PMC5397495

[ref2] Alippe Y. Hauk V. Mor G. Vota D. M. (2025). Editorial: host-pathogen interactions during pregnancy: mechanisms of maternal and fetal immunity. Front. Immunol. 16:1629528. doi: 10.3389/fimmu.2025.1629528, 40557141 PMC12185400

[ref3] Aljohni M. S. Harun-Ur-Rashid M. Selim S. (2025). Emerging threats: antimicrobial resistance in extended-spectrum beta-lactamase and carbapenem-resistant *Escherichia coli*. Microb. Pathog. 200:107275. doi: 10.1016/j.micpath.2024.107275, 39798725

[ref4] Alkeskas A. Ogrodzki P. Saad M. Masood N. Rhoma N. R. Moore K. . (2015). The molecular characterisation of *Escherichia coli* K1 isolated from neonatal nasogastric feeding tubes. BMC Infect. Dis. 15:449. doi: 10.1186/s12879-015-1210-7, 26497222 PMC4620641

[ref5] Al-Mayahie S. M. (2013a). Phenotypic and genotypic comparison of ESBL production by vaginal *Escherichia coli* isolates from pregnant and non-pregnant women. Ann. Clin. Microbiol. Antimicrob. 12:7. doi: 10.1186/1476-0711-12-7, 23617811 PMC3661376

[ref6] Al-Mayahie S. M. (2013b). Vaginal colonization by papG allele II+ *Escherichia coli* isolates from pregnant and nonpregnant women as predisposing factor to pyelonephritis. Infect. Dis. Obstet. Gynecol. 2013:860402. doi: 10.1155/2013/860402, 23861574 PMC3703789

[ref7] Amabebe E. Anumba D. O. C. (2018). The vaginal microenvironment: the physiologic role of lactobacilli. Front. Med. 5:181. doi: 10.3389/fmed.2018.00181, 29951482 PMC6008313

[ref8] Amabebe E. Anumba D. O. C. (2020). Female gut and genital tract microbiota-induced crosstalk and differential effects of short-chain fatty acids on immune sequelae. Front. Immunol. 11:2184. doi: 10.3389/fimmu.2020.02184, 33013918 PMC7511578

[ref9] Antoine C. Laforêt F. Goya-Jorge E. Gonza I. Lebrun S. Douny C. . (2023). Phage targeting neonatal Meningitis *E. coli* K1 in vitro in the intestinal microbiota of pregnant donors and impact on bacterial populations. Int. J. Mol. Sci. 24:580. doi: 10.3390/ijms241310580, 37445758 PMC10341584

[ref10] Arredondo-Alonso S. Blundell-Hunter G. Fu Z. Gladstone R. A. Fillol-Salom A. Loraine J. . (2023). Evolutionary and functional history of the *Escherichia coli* K1 capsule. Nat. Commun. 14:3294. doi: 10.1038/s41467-023-39052-w, 37322051 PMC10272209

[ref11] Asgedom S. G. Gebrewahd D. T. Abdi M. G. Wondie Y. A. Melese E. A. Belete K. T. (2025). Management of rare intraventricular empyema secondary to late onset neonatal meningitis in an infant: A case report. Int. J. Surg. Case Rep. 134:111791. doi: 10.1016/j.ijscr.2025.111791, 40803251 PMC12361597

[ref12] Balla B. Illés A. Tobiás B. Pikó H. Beke A. Sipos M. . (2024). The role of the vaginal and endometrial microbiomes in infertility and their impact on pregnancy outcomes in light of recent literature. Int. J. Mol. Sci. 25:227. doi: 10.3390/ijms252313227, 39684937 PMC11642076

[ref13] Basmaci R. Bonacorsi S. Bidet P. Biran V. Aujard Y. Bingen E. . (2015). *Escherichia Coli* meningitis features in 325 children from 2001 to 2013 in France. Clin. Infect. Dis. 61, 779–786. doi: 10.1093/cid/civ367, 25944342

[ref14] Bauserman M. S. Laughon M. M. Hornik C. P. Smith P. B. Benjamin D. K. Jr. Clark R. H. . (2013). Group B *Streptococcus* and *Escherichia coli* infections in the intensive care nursery in the era of intrapartum antibiotic prophylaxis. Pediatr. Infect. Dis. J. 32, 208–212. doi: 10.1097/INF.0b013e318275058a, 23011013 PMC3572304

[ref15] Bhagirath A. Y. Medapati M. R. de Jesus V. C. Yadav S. Hinton M. Dakshinamurti S. . (2021). Role of maternal infections and inflammatory responses on craniofacial development. Front. Oral Health 2:735634. doi: 10.3389/froh.2021.735634, 35048051 PMC8757860

[ref16] Bhattacharjee A. Sands K. Mitra S. Basu R. Saha B. Clermont O. . (2023). A decade-long evaluation of neonatal Septicaemic *Escherichia coli*: clonal lineages, genomes, and New Delhi Metallo-Beta-lactamase variants. Microbiol. Spectrum 11:e0521522. doi: 10.1128/spectrum.05215-22, 37367488 PMC10434172

[ref17] Biggel M. Xavier B. B. Johnson J. R. Nielsen K. L. Frimodt-Møller N. Matheeussen V. . (2020). Horizontally acquired papGII-containing pathogenicity islands underlie the emergence of invasive uropathogenic *Escherichia coli* lineages. Nat. Commun. 11:5968. doi: 10.1038/s41467-020-19714-9, 33235212 PMC7686366

[ref18] Bingen E. Lambert-Zechovsky N. Mariani-Kurkdjian P. Doit C. Aujard Y. Fournerie F. . (1990). Bacterial counts in cerebrospinal fluid of children with meningitis. Eur. J. Clin. Microbiol. Infect. Dis. 9, 278–281. doi: 10.1007/bf01968060, 2112465

[ref19] Biran D. Ron E. Z. (2018). Extraintestinal pathogenic *Escherichia coli*. Curr. Top. Microbiol. Immunol. 416, 149–161. doi: 10.1007/82_2018_108, 30046982

[ref20] Biran D. Sura T. Otto A. Yair Y. Becher D. Ron E. Z. (2021). Surviving serum: the *Escherichia coli* iss gene of Extraintestinal Pathogenic *E. coli* is required for the synthesis of group 4 capsule. Infect. Immun. 89:e0031621. doi: 10.1128/iai.00316-21, 34181459 PMC8445191

[ref21] Birhane Fiseha S. Mulatu Jara G. Azerefegn Woldetsadik E. Belayneh Bekele F. Mohammed Ali M. (2021). Colonization rate of potential neonatal disease-causing bacteria, associated factors, and antimicrobial susceptibility profile among pregnant women attending government hospitals in Hawassa, Ethiopia. Infect. Drug Resist. 14, 3159–3168. doi: 10.2147/idr.S326200, 34429615 PMC8374838

[ref22] Bizuwork K. Alemayehu H. Medhin G. Amogne W. Eguale T. (2021). Asymptomatic bacteriuria among pregnant women in Addis Ababa, Ethiopia: prevalence, causal agents, and their antimicrobial susceptibility. Int J Microbiol. 2021, 1–8. doi: 10.1155/2021/8418043, 34335781 PMC8313335

[ref23] Bok E. Kożańska A. Mazurek-Popczyk J. Wojciech M. Baldy-Chudzik K. (2020). Extended phylogeny and Extraintestinal virulence potential of commensal *Escherichia coli* from piglets and sows. Int. J. Environ. Res. Public Health 17:366. doi: 10.3390/ijerph17010366, 31935799 PMC6981902

[ref24] Bonacorsi S. Bingen E. (2005). Molecular epidemiology of *Escherichia coli* causing neonatal meningitis. Int. J. Med. Microbiol. 295, 373–381. doi: 10.1016/j.ijmm.2005.07.011, 16238014

[ref25] Boutouchent N. Vu T. N. A. Landraud L. Kennedy S. P. (2024). Urogenital colonization and pathogenicity of *E. coli* in the vaginal microbiota during pregnancy. Sci. Rep. 14:25523. doi: 10.1038/s41598-024-76438-2, 39462143 PMC11513020

[ref26] Brokaw A. Furuta A. Dacanay M. Rajagopal L. Adams Waldorf K. M. (2021). Bacterial and host determinants of group B streptococcal vaginal colonization and ascending infection in pregnancy. Front. Cell. Infect. Microbiol. 11:720789. doi: 10.3389/fcimb.2021.720789, 34540718 PMC8446444

[ref27] Brown R. G. Al-Memar M. Marchesi J. R. Lee Y. S. Smith A. Chan D. . (2019). Establishment of vaginal microbiota composition in early pregnancy and its association with subsequent preterm prelabor rupture of the fetal membranes. Transl. Res. 207:30633889, 30–43. doi: 10.1016/j.trsl.2018.12.005 PMC6489901 30633889

[ref28] Cadieux P. A. Burton J. Devillard E. Reid G. (2009). *Lactobacillus* by-products inhibit the growth and virulence of uropathogenic *Escherichia coli*. J. Physiol. Pharmacol. 60, 13–18, 20224146

[ref29] Cappelletti M. Presicce P. Kallapur S. G. (2020). Immunobiology of acute chorioamnionitis. Front. Immunol. 11:649. doi: 10.3389/fimmu.2020.00649, 32373122 PMC7177011

[ref30] Carlini F. Maroccia Z. Fiorentini C. Travaglione S. Fabbri A. (2021). Effects of the *Escherichia coli* bacterial toxin cytotoxic necrotizing factor 1 on different human and animal cells: A systematic review. Int. J. Mol. Sci. 22:610. doi: 10.3390/ijms222212610, 34830494 PMC8621085

[ref31] Carter S. W. D. Neubronner S. Su L. L. Dashraath P. Mattar C. Illanes S. E. . (2023). Chorioamnionitis: An update on diagnostic evaluation. Biomedicine 11:922. doi: 10.3390/biomedicines11112922, 38001923 PMC10669668

[ref32] CDC. (2019). Antibiotic Resistance Threats in the United States. Atlanta, GA: U.S. Department of Health and Human Services.

[ref33] CDC. (2024). Early-onset neonatal Sepsis surveillance and trends. Atlanta, GA: Centers for Disease Control and Prevention.

[ref34] Celik I. H. Hanna M. Canpolat F. E. Mohan P. (2022). Diagnosis of neonatal sepsis: the past, present and future. Pediatr. Res. 91, 337–350. doi: 10.1038/s41390-021-01696-z, 34728808 PMC8818018

[ref35] Chan C. Ng D. Fraser M. E. Schryvers A. B. (2023). Structural and functional insights into iron acquisition from lactoferrin and transferrin in gram-negative bacterial pathogens. Biometals 36, 683–702. doi: 10.1007/s10534-022-00466-6, 36418809 PMC10182148

[ref36] Chaurasia S. Sivanandan S. Agarwal R. Ellis S. Sharland M. Sankar M. J. (2019). Neonatal sepsis in South Asia: huge burden and spiralling antimicrobial resistance. BMJ 364:k5314. doi: 10.1136/bmj.k5314, 30670451 PMC6340339

[ref37] Choi Y. S. Kim Y. Hong S. Y. Cho H. J. Sung J. H. Choi S. J. . (2023a). Abnormal vaginal Flora in cervical incompetence patients - the impact of *Escherichia coli*. Reprod. Sci. 30, 3010–3018. doi: 10.1007/s43032-023-01242-8, 37118059

[ref38] Choi Y. S. Kim J. H. Kim Y. Cho H. J. Sung J. H. Choi S. J. . (2023b). Growing threat of extended-spectrum β-lactamase-producing Enterobacteriaceae colonisation in high-risk pregnancies: A cross-sectional study. BJOG 130, 415–423. doi: 10.1111/1471-0528.17194, 35445798

[ref39] Cieza R. J. Hu J. Ross B. N. Sbrana E. Torres A. G. (2015). The IbeA invasin of adherent-invasive *Escherichia coli* mediates interaction with intestinal epithelia and macrophages. Infect. Immun. 83, 1904–1918. doi: 10.1128/iai.03003-14, 25712929 PMC4399045

[ref40] Clark J. R. Maresso A. M. (2021). Comparative Pathogenomics of *Escherichia coli*: polyvalent vaccine target identification through Virulome analysis. Infect. Immun. 89:e0011521. doi: 10.1128/iai.00115-21, 33941580 PMC8281228

[ref41] Cole B. K. Ilikj M. McCloskey C. B. Chavez-Bueno S. (2019). Antibiotic resistance and molecular characterization of bacteremia *Escherichia coli* isolates from newborns in the United States. PLoS One 14:e0219352. doi: 10.1371/journal.pone.0219352, 31276562 PMC6611611

[ref42] da Silva L. C. de Mello Santos A. C. Silva R. M. (2017). Uropathogenic *Escherichia coli* pathogenicity islands and other ExPEC virulence genes may contribute to the genome variability of enteroinvasive *E. coli*. BMC Microbiol. 17:68. doi: 10.1186/s12866-017-0979-5, 28302076 PMC5356261

[ref43] Dale A. P. Woodford N. (2015). Extra-intestinal pathogenic *Escherichia coli* (ExPEC): disease, carriage and clones. J. Infect. 71, 615–626. doi: 10.1016/j.jinf.2015.09.009, 26409905

[ref44] Day M. J. Hopkins K. L. Wareham D. W. Toleman M. A. Elviss N. Randall L. . (2019). Extended-spectrum β-lactamase-producing *Escherichia coli* in human-derived and foodchain-derived samples from England, Wales, and Scotland: an epidemiological surveillance and typing study. Lancet Infect. Dis. 19, 1325–1335. doi: 10.1016/s1473-3099(19)30273-7, 31653524

[ref45] de As C. Dias L. Cardona R. S. B. Variane G. F. T. do Nascimento S. D. de Oliveira S. R. P. . (2020). Anaerobic neonatal meningitis: a diagnostic challenge. Anaerobe 61:102134. doi: 10.1016/j.anaerobe.2019.102134 31838318

[ref46] De Francesco M. A. Bertelli A. Corbellini S. Scaltriti E. Risso F. Allegri R. . (2023). Emergence of pandemic clonal lineage sequence types 131 and 69 of extraintestinal *Escherichia coli* as a cause of meningitis: is it time to revise molecular assays? Microbiol. Spectrum 11:e0327422. doi: 10.1128/spectrum.03274-22, 36786647 PMC10100906

[ref47] Deress T. Belay G. Ayenew G. Ferede W. Worku M. Feleke T. . (2024). Bacterial profiles and their antibiotic susceptibility patterns in neonatal sepsis at the University of Gondar Comprehensive Specialized Hospital, Ethiopia. Front. Microbiol. 15:1461689. doi: 10.3389/fmicb.2024.1461689, 39498130 PMC11532188

[ref48] Desvaux M. Dalmasso G. Beyrouthy R. Barnich N. Delmas J. Bonnet R. (2020). Pathogenicity factors of Genomic Islands in intestinal and Extraintestinal *Escherichia coli*. Front. Microbiol. 11:2065. doi: 10.3389/fmicb.2020.02065, 33101219 PMC7545054

[ref49] Dhakal B. K. Mulvey M. A. (2012). The UPEC pore-forming toxin α-hemolysin triggers proteolysis of host proteins to disrupt cell adhesion, inflammatory, and survival pathways. Cell Host Microbe 11, 58–69. doi: 10.1016/j.chom.2011.12.003, 22264513 PMC3266558

[ref50] Diep R. E. Adhikari U. Gokce Tezel K. Pham G. Burrell A. R. Staat M. A. . (2026). Natural maternal immunity protects neonates from *Escherichia coli* sepsis. Nature 653, 519–527. doi: 10.1038/s41586-026-10225-z, 41813901 PMC13108393

[ref51] Doenhardt M. Seipolt B. Mense L. Winkler J. L. Thürmer A. Rüdiger M. . (2020). Neonatal and young infant sepsis by group B streptococci and *Escherichia coli*: a single-center retrospective analysis in Germany-GBS screening implementation gaps and reduction in antibiotic resistance. Eur. J. Pediatr. 179, 1769–1777. doi: 10.1007/s00431-020-03659-8, 32447562 PMC7547982

[ref52] Eldridge G. R. Hughey H. Rosenberger L. Martin S. M. Shapiro A. M. D'Antonio E. . (2021). Safety and immunogenicity of an adjuvanted *Escherichia coli* adhesin vaccine in healthy women with and without histories of recurrent urinary tract infections: results from a first-in-human phase 1 study. Hum. Vaccin. Immunother. 17, 1262–1270. doi: 10.1080/21645515.2020.1834807 33325785 PMC8078672

[ref53] Flannery D. D. Akinboyo I. C. Mukhopadhyay S. Tribble A. C. Song L. Chen F. . (2021). Antibiotic susceptibility of *Escherichia coli* among infants admitted to neonatal intensive care units across the US from 2009 to 2017. JAMA Pediatr. 175, 168–175. doi: 10.1001/jamapediatrics.2020.4719, 33165599 PMC7653538

[ref54] Flannery D. D. Chiotos K. Gerber J. S. Puopolo K. M. (2022a). Neonatal multidrug-resistant gram-negative infection: epidemiology, mechanisms of resistance, and management. Pediatr. Res. 91, 380–391. doi: 10.1038/s41390-021-01745-7, 34599280 PMC8819496

[ref55] Flannery D. D. Puopolo K. M. Hansen N. I. Gerber J. S. Sánchez P. J. Stoll B. J. (2022b). Antimicrobial susceptibility profiles among neonatal early-onset Sepsis pathogens. Pediatr. Infect. Dis. J. 41, 263–271. doi: 10.1097/inf.0000000000003380, 34862339 PMC8831448

[ref56] Fleischmann-Struzek C. Goldfarb D. M. Schlattmann P. Schlapbach L. J. Reinhart K. Kissoon N. (2018). The global burden of paediatric and neonatal sepsis: a systematic review. Lancet Respir. Med. 6, 223–230. doi: 10.1016/s2213-2600(18)30063-8, 29508706

[ref57] Fleiss N. Schwabenbauer K. Randis T. M. Polin R. A. (2023). What's new in the management of neonatal early-onset sepsis? Arch. Dis. Child. Fetal Neonatal Ed. 108, 10–14. doi: 10.1136/archdischild-2021-323532, 35618407

[ref58] Frank Wolf M. Abu Shqara R. Naskovica K. Zilberfarb I. A. Sgayer I. Glikman D. . (2021). Vertical transmission of extended-Spectrum, Beta-lactamase-producing Enterobacteriaceae during preterm delivery: A prospective study. Microorganisms 9:506. doi: 10.3390/microorganisms9030506, 33673648 PMC7997221

[ref59] Frenck R. W. Jr. Ervin J. Chu L. Abbanat D. Spiessens B. Go O. . (2019). Safety and immunogenicity of a vaccine for extra-intestinal pathogenic *Escherichia coli* (ESTELLA): a phase 2 randomised controlled trial. Lancet Infect. Dis. 19, 631–640. doi: 10.1016/s1473-3099(18)30803-x, 31079947

[ref60] Gad A. Alkhdr M. Terkawi R. Alsharif H. Ibrahim M. Amin R. . (2024). Associations between maternal bacteremia during the peripartum period and early-onset neonatal sepsis: a retrospective cohort study. BMC Pediatr. 24:526. doi: 10.1186/s12887-024-04980-z, 39143544 PMC11325601

[ref61] Galardini M. Clermont O. Baron A. Busby B. Dion S. Schubert S. . (2020). Major role of iron uptake systems in the intrinsic extra-intestinal virulence of the genus Escherichia revealed by a genome-wide association study. PLoS Genet. 16:e1009065. doi: 10.1371/journal.pgen.1009065, 33112851 PMC7592755

[ref62] García F. G. Li Y. Vassar R. Hawkins C. Petersen M. Gano D. (2024). Cerebrovascular injury from early-onset neonatal *Escherichia coli* meningitis: expanding the clinical-radiologic phenotype. Pediatr. Neurol. 161, 167–169. doi: 10.1016/j.pediatrneurol.2024.09.020, 39388736

[ref63] Garcia T. A. Ventura C. L. Smith M. A. Merrell D. S. O'Brien A. D. (2013). Cytotoxic necrotizing factor 1 and hemolysin from uropathogenic *Escherichia coli* elicit different host responses in the murine bladder. Infect. Immun. 81, 99–109. doi: 10.1128/iai.00605-12, 23090961 PMC3536159

[ref64] Gaschignard J. Levy C. Romain O. Cohen R. Bingen E. Aujard Y. . (2011). Neonatal bacterial meningitis: 444 cases in 7 years. Pediatr. Infect. Dis. J. 30, 212–217. doi: 10.1097/INF.0b013e3181fab1e7, 21416693

[ref65] Ghartey J. P. Smith B. C. Chen Z. Buckley N. Lo Y. Ratner A. J. . (2014). *Lactobacillus crispatus* dominant vaginal microbiome is associated with inhibitory activity of female genital tract secretions against *Escherichia coli*. PLoS One 9:e96659. doi: 10.1371/journal.pone.0096659, 24805362 PMC4013016

[ref66] Gomez-Lopez N. Galaz J. Miller D. Farias-Jofre M. Liu Z. Arenas-Hernandez M. . (2022). The immunobiology of preterm labor and birth: intra-amniotic inflammation or breakdown of maternal-fetal homeostasis. Reproduction 164, R11–R45. doi: 10.1530/rep-22-0046, 35559791 PMC9233101

[ref67] Grome H. N. Brandenburg J. M. Kent A. G. Curtis L. Raymond R. E. Ansari U. . (2026). Extraintestinal invasive *Escherichia coli* infections in the US. JAMA Netw. Open 9:e2557201. doi: 10.1001/jamanetworkopen.2025.57201, 41627814 PMC12865657

[ref68] Guiral E. Bosch J. Vila J. Soto S. M. (2011). Prevalence of *Escherichia coli* among samples collected from the genital tract in pregnant and nonpregnant women: relationship with virulence. FEMS Microbiol. Lett. 314, 170–173. doi: 10.1111/j.1574-6968.2010.02160.x, 21133987

[ref69] Guiral E. Sáez-López E. Bosch J. Goncé A. López M. Sanz S. . (2015). Antimicrobial resistance and virulence characterization among *Escherichia coli* clinical isolates causing severe obstetric infections in pregnant women. J. Clin. Microbiol. 53, 1745–1747. doi: 10.1128/jcm.00487-15, 25740771 PMC4400784

[ref70] He X. T. Chang C. N. Yu C. H. Wang C. C. (2024). The risk factors, antimicrobial resistance patterns, and outcomes associated with extended-spectrum β-lactamases-producing pathogens in pediatric urinary tract infection. Pediatr. Neonatol. 65, 242–248. doi: 10.1016/j.pedneo.2023.04.021, 37951832

[ref71] Hilbert D. W. Paulish T. E. Mordechai E. Adelson M. E. Trama J. P. (2008). O serogroups, phylogeny, and virulence factors of cervicovaginal and rectal *Escherichia coli* isolates. Eur. J. Clin. Microbiol. Infect. Dis. 27, 1265–1268. doi: 10.1007/s10096-008-0574-7, 18584221

[ref72] Hoffman A. Satyavolu S. Muhanna D. Malay S. Raffay T. Windau A. . (2024). Predictors of mortality and severe illness from *Escherichia coli* sepsis in neonates. J. Perinatol. 44, 1816–1821. doi: 10.1038/s41372-024-02117-9, 39266664 PMC11606913

[ref73] Hsu M. H. Hsu J. F. Kuo H. C. Lai M. Y. Chiang M. C. Lin Y. J. . (2018). Neurological complications in young infants with acute bacterial meningitis. Front. Neurol. 9:903. doi: 10.3389/fneur.2018.00903, 30405525 PMC6207629

[ref74] Huang S. H. Stins M. F. Kim K. S. (2000). Bacterial penetration across the blood-brain barrier during the development of neonatal meningitis. Microbes Infect. 2, 1237–1244. doi: 10.1016/s1286-4579(00)01277-6, 11008113

[ref75] Huang S. H. Wan Z. S. Chen Y. H. Jong A. Y. Kim K. S. (2001). Further characterization of *Escherichia coli* brain microvascular endothelial cell invasion gene ibeA by deletion, complementation, and protein expression. J. Infect. Dis. 183, 1071–1078. doi: 10.1086/319290, 11237832

[ref76] Hudson P. L. Hung K. J. Bergerat A. Mitchell C. (2020). Effect of vaginal Lactobacillus species on *Escherichia coli* growth. Female Pelvic Med. Reconstr. Surg. 26, 146–151. doi: 10.1097/spv.0000000000000827, 31990804

[ref77] Huttner A. Hatz C. van den Dobbelsteen G. Abbanat D. Hornacek A. Frölich R. . (2017). Safety, immunogenicity, and preliminary clinical efficacy of a vaccine against extraintestinal pathogenic *Escherichia coli* in women with a history of recurrent urinary tract infection: a randomised, single-blind, placebo-controlled phase 1b trial. Lancet Infect. Dis. 17, 528–537. doi: 10.1016/s1473-3099(17)30108-1, 28238601

[ref78] Hwang J. Kim S. Kim H. Kim C. Kim S. H. Yang M. . (2024). Predictive factors for perinatal bacterial transmission from colonized mothers to delivered very-low-birth-weight infants: a retrospective cohort study. Sci. Rep. 14:16835. doi: 10.1038/s41598-024-67674-7, 39039134 PMC11263601

[ref79] Inoue M. Ogawa T. Tamura H. Hagiwara Y. Saito Y. Abbanat D. . (2018). Safety, tolerability and immunogenicity of the ExPEC4V (JNJ-63871860) vaccine for prevention of invasive extraintestinal pathogenic *Escherichia coli* disease: a phase 1, randomized, double-blind, placebo-controlled study in healthy Japanese participants. Hum. Vaccin. Immunother. 14, 2150–2157. doi: 10.1080/21645515.2018.1474316, 29771596 PMC6183137

[ref80] Joshi N. S. Huynh K. Lu T. Lee H. C. Frymoyer A. (2022). Epidemiology and trends in neonatal early onset sepsis in California, 2010-2017. J. Perinatol. 42, 940–946. doi: 10.1038/s41372-022-01393-7, 35469043

[ref81] Kaczmarek A. Skowron K. Budzyńska A. Gospodarek-Komkowska E. (2018). Virulence-associated genes and antibiotic susceptibility among vaginal and rectal *Escherichia coli* isolates from healthy pregnant women in Poland. Folia Microbiol. 63, 637–643. doi: 10.1007/s12223-018-0598-z, 29644511 PMC6097025

[ref82] Kang D. Kirienko N. V. (2018). Interdependence between iron acquisition and biofilm formation in *Pseudomonas aeruginosa*. J. Microbiol. 56, 449–457. doi: 10.1007/s12275-018-8114-3, 29948830 PMC6221862

[ref83] Kariniotaki C. Thomou C. Gkentzi D. Panteris E. Dimitriou G. Hatzidaki E. (2024). Neonatal sepsis: a comprehensive review. Antibiotics 14. doi: 10.3390/antibiotics14010006, 39858292 PMC11761862

[ref84] Kocsis B. Gulyás D. Szabó D. (2022). Emergence and dissemination of Extraintestinal pathogenic high-risk international clones of *Escherichia coli*. Life 12:77. doi: 10.3390/life12122077, 36556442 PMC9780897

[ref85] Koizumi A. Maruyama K. Ohki Y. Nakayama A. Yamada Y. Kurosawa H. . (2020). Prevalence and risk factor for antibiotic-resistant *Escherichia coli* colonization at birth in premature infants: A prospective cohort study. Pediatr. Infect. Dis. J. 39, 546–552. doi: 10.1097/inf.0000000000002623, 32118857

[ref86] Korang S. K. Safi S. Nava C. Greisen G. Gupta M. Lausten-Thomsen U. . (2021). Antibiotic regimens for late-onset neonatal sepsis. Cochrane Database Syst. Rev. 2021:Cd013836. doi: 10.1002/14651858.CD013836.pub2, 33998665 PMC8127057

[ref87] Lai J. Zhu Y. Tang L. Lin X. (2021). Epidemiology and antimicrobial susceptibility of invasive *Escherichia coli* infection in neonates from 2012 to 2019 in Xiamen, China. BMC Infect. Dis. 21:295. doi: 10.1186/s12879-021-05981-4, 33757434 PMC7988952

[ref88] Leroux-Roels I. Day T. A. Deleu S. McLean C. Go O. Davies T. A. . (2026). Immunogenicity and safety of the ExPEC9V *Escherichia coli* vaccine co-administered with a high-dose influenza vaccine in older adults: a placebo-controlled, randomized, phase 3 study. Vaccine 14:146. doi: 10.3390/vaccines14020146, 41746067 PMC12944986

[ref89] Liu T. H. Wang H. P. Cho F. N. Wang J. L. Hung C. H. Chiou Y. H. . (2019). Rectovaginal colonization with pathogenic *Escherichia coli* during pregnancy and neonatal outcomes. Infect. Drug Resist. 12, 3103–3112. doi: 10.2147/idr.S207857, 31686871 PMC6777437

[ref90] Liu Y. Zhu M. Fu X. Cai J. Chen S. Lin Y. . (2021). *Escherichia coli* causing neonatal meningitis during 2001-2020: A study in eastern China. Int. J. Gen. Med. 14, 3007–3016. doi: 10.2147/ijgm.S317299, 34234530 PMC8254664

[ref91] Lu I. C. Chang Y. C. Chen Y. T. Lin H. Y. Chiu H. Y. Tsai M. L. . (2022). Epidemiological evolution of early-onset neonatal sepsis over 12 years: a single center, population-based study in Central Taiwan. J. Neonatal. Perinatal. Med. 15, 575–582. doi: 10.3233/npm-210938, 35404292

[ref92] Lukanović D. Batkoska M. Kavšek G. Druškovič M. (2023). Clinical chorioamnionitis: where do we stand now? Front. Med. 10:1191254. doi: 10.3389/fmed.2023.1191254, 37293298 PMC10244675

[ref93] Lungu N. Jura A. M. Popescu D. E. Horhat F. G. Manea A. M. Boia M. (2024). Understanding the difficulties in diagnosing neonatal Sepsis: assessing the role of Sepsis biomarkers. J. Crit. Care Med. 10, 316–328. doi: 10.2478/jccm-2024-0039, 39829727 PMC11740700

[ref94] Ma J. An C. Jiang F. Yao H. Logue C. Nolan L. K. . (2018). Extraintestinal pathogenic *Escherichia coli* increase extracytoplasmic polysaccharide biosynthesis for serum resistance in response to bloodstream signals. Mol. Microbiol. 110, 689–706. doi: 10.1111/mmi.13987, 29802751

[ref95] Malaure C. Geslain G. Birgy A. Bidet P. Poilane I. Allain M. . (2024). Early-onset infection caused by *Escherichia coli* sequence type 1193 in late preterm and full-term neonates. Emerg. Infect. Dis. 30, 20–28. doi: 10.3201/eid3001.230851, 38146959 PMC10756391

[ref96] Mamo B. T. Bonger Z. T. Senbato F. R. Eguale T. Akililu K. K. Welelaw S. M. . (2025). Gram-negative bacterial sepsis, antimicrobial susceptibility pattern and treatment outcomes at two neonatal intensive care units in Addis Ababa, Ethiopia: A retrospective observational study. PLoS One 20:e0323288. doi: 10.1371/journal.pone.0323288, 40359214 PMC12074502

[ref97] Mao Z. Zhang H. Cai W. Yang Y. Zhang X. Jiang F. . (2023). NhaA facilitates the maintenance of bacterial envelope integrity and the evasion of complement attack contributing to extraintestinal pathogenic *Escherichia coli* virulence. Infect. Immun. 91:e0003923. doi: 10.1128/iai.00039-23, 37815368 PMC10652942

[ref98] Maruvada R. Kim K. S. (2012). IbeA and OmpA of *Escherichia coli* K1 exploit Rac1 activation for invasion of human brain microvascular endothelial cells. Infect. Immun. 80, 2035–2041. doi: 10.1128/iai.06320-11, 22451524 PMC3370590

[ref99] Massella E. Giacometti F. Bonilauri P. Reid C. J. Djordjevic S. P. Merialdi G. . (2021). Antimicrobial resistance profile and ExPEC virulence potential in commensal *Escherichia coli* of multiple sources. Antibiotics 10:351. doi: 10.3390/antibiotics10040351, 33810387 PMC8067153

[ref100] Mathias S. North K. Santana A. Britto C. Fung A. Chou R. . (2024). Efficacy of antibiotic regimens for meningitis in young infants aged 0-59 days: a systematic review. Pediatrics 154:88H. doi: 10.1542/peds.2024-066588H, 39087804

[ref101] Meena P. R. Priyanka P. Singh A. P. (2023). Extraintestinal pathogenic *Escherichia coli* (ExPEC) reservoirs, and antibiotics resistance trends: a one-health surveillance for risk analysis from "farm-to-fork". Lett. Appl. Microbiol. 76:16. doi: 10.1093/lambio/ovac016, 36688760

[ref102] Megli C. J. Coyne C. B. (2022). Infections at the maternal-fetal interface: an overview of pathogenesis and defence. Nat. Rev. Microbiol. 20, 67–82. doi: 10.1038/s41579-021-00610-y, 34433930 PMC8386341

[ref103] Mendoza-Palomar N. Balasch-Carulla M. González-Di Lauro S. Céspedes M. C. Andreu A. Frick M. A. . (2017). *Escherichia coli* early-onset sepsis: trends over two decades. Eur. J. Pediatr. 176, 1227–1234. doi: 10.1007/s00431-017-2975-z, 28770413

[ref104] Messerer M. Fischer W. Schubert S. (2017). Investigation of horizontal gene transfer of pathogenicity islands in *Escherichia coli* using next-generation sequencing. PLoS One 12:e0179880. doi: 10.1371/journal.pone.0179880, 28732043 PMC5521745

[ref105] Michels K. R. Lambrecht N. J. Carson W. F. Schaller M. A. Lukacs N. W. Bermick J. R. (2019). The role of Iron in the susceptibility of neonatal mice to *Escherichia coli* K1 Sepsis. J. Infect. Dis. 220, 1219–1229. doi: 10.1093/infdis/jiz282, 31136646 PMC7325330

[ref106] Miselli F. Cuoghi Costantini R. Creti R. Sforza F. Fanaro S. Ciccia M. . (2022). *Escherichia coli* is overtaking group B Streptococcus in early-onset neonatal Sepsis. Microorganisms 10:878. doi: 10.3390/microorganisms10101878, 36296155 PMC9607315

[ref107] Mohamed F. Y. Dahie H. A. Mohamoud J. H. Adam M. H. Dirie H. M. (2023). Prevalence, antimicrobial susceptibility profile, and associated risk factors of uropathogenic *Escherichia coli* among pregnant women attending Dr. Sumait hospital Mogadishu, Somalia. Front. Public Health 11:1203913. doi: 10.3389/fpubh.2023.1203913, 38328535 PMC10847321

[ref108] Moradi Y. Eshrati B. Motevalian S. A. Majidpour A. Baradaran H. R. (2021). A systematic review and meta-analysis on the prevalence of Escherichia coli and extended-spectrum β-lactamase-producing *Escherichia coli* in pregnant women. Arch. Gynecol. Obstet. 303, 363–379. doi: 10.1007/s00404-020-05903-w, 33386957

[ref109] Motomura K. Romero R. Galaz J. Tarca A. L. Done B. Xu Y. . (2021). RNA sequencing reveals distinct immune responses in the Chorioamniotic membranes of women with preterm labor and microbial or sterile intra-amniotic inflammation. Infect. Immun. 89:20. doi: 10.1128/iai.00819-20, 33558326 PMC8091089

[ref110] Murdoch C. C. Skaar E. P. (2022). Nutritional immunity: the battle for nutrient metals at the host-pathogen interface. Nat. Rev. Microbiol. 20, 657–670. doi: 10.1038/s41579-022-00745-6, 35641670 PMC9153222

[ref111] Nagarjuna D. Mittal G. Dhanda R. S. Verma P. K. Gaind R. Yadav M. (2015). Faecal *Escherichia coli* isolates show potential to cause endogenous infection in patients admitted to the ICU in a tertiary care hospital. New Microbes New Infect. 7, 57–66. doi: 10.1016/j.nmni.2015.05.006, 26257914 PMC4522595

[ref112] Negishi Y. Shima Y. Kato M. Ichikawa T. Ino H. Horii Y. . (2022). Inflammation in preterm birth: novel mechanism of preterm birth associated with innate and acquired immunity. J. Reprod. Immunol. 154:103748. doi: 10.1016/j.jri.2022.103748, 36126439

[ref113] Nhu N. T. K. Phan M. D. Hancock S. J. Peters K. M. Alvarez-Fraga L. Forde B. M. . (2024). High-risk *Escherichia coli* clones that cause neonatal meningitis and association with recrudescent infection. eLife 12:12. doi: 10.7554/eLife.91853, 38622998 PMC11021048

[ref114] Nishihara Y. Zaniletti I. Zenge J. Weikel B. Parker S. Murthy K. . (2025). Epidemiology of bacterial and fungal infections among level IV neonatal units in North America. J. Perinatol. 45, 997–1004. doi: 10.1038/s41372-025-02337-7, 40542124

[ref115] Oldendorff F. Linnér A. Finder M. Eisenlauer P. Kjellberg M. Giske C. G. . (2022). Case report: fatal outcome for a preterm newborn with meningitis caused by extended-Spectrum β-lactamase-producing *Escherichia coli* sequence type 1193. Front. Pediatr. 10:866762. doi: 10.3389/fped.2022.866762, 35463903 PMC9019577

[ref116] Oliveira F. Lima T. Correia A. Silva A. M. Soares C. Morais S. . (2021). Siderophore-mediated Iron acquisition plays a critical role in biofilm formation and survival of *Staphylococcus epidermidis* within the host. Front. Med. 8:799227. doi: 10.3389/fmed.2021.799227, 35004774 PMC8738164

[ref117] Olmos-Ortiz A. Hernández-Pérez M. Flores-Espinosa P. Sedano G. Helguera-Repetto A. C. Villavicencio-Carrisoza Ó. . (2022). Compartmentalized innate immune response of human fetal membranes against *Escherichia coli* Choriodecidual infection. Int. J. Mol. Sci. 23:94. doi: 10.3390/ijms23062994, 35328414 PMC8949057

[ref118] Ouchenir L. Renaud C. Khan S. Bitnun A. Boisvert A. A. McDonald J. . (2017). The epidemiology, management, and outcomes of bacterial meningitis in infants. Pediatrics 140:76. doi: 10.1542/peds.2017-0476, 28600447

[ref119] Park S. So H. Kim M. N. Lee J. (2022). Initial empirical antibiotics of non-carbapenems for ESBL-producing E. Coli and *K. pneumoniae* bacteremia in children: a retrospective medical record review. BMC Infect. Dis. 22:866. doi: 10.1186/s12879-022-07881-7, 36404302 PMC9677890

[ref120] Pearse O. Zuza A. Tewesa E. Siyabu P. Fraser A. J. Cornick J. . (2025). High diversity of *Escherichia coli* causing invasive disease in neonates in Malawi poses challenges for O-antigen based vaccine approach. Commun. Med. (Lond.) 5:298. doi: 10.1038/s43856-025-01007-1, 40681780 PMC12274568

[ref121] Pitout J. D. D. Peirano G. Chen L. DeVinney R. Matsumura Y. (2022). *Escherichia coli* ST1193: following in the footsteps of *E. coli* ST131. Antimicrob. Agents Chemother. 66:e0051122. doi: 10.1128/aac.00511-22, 35658504 PMC9295538

[ref122] Poolman J. T. Wacker M. (2016). Extraintestinal pathogenic *Escherichia coli*, a common human pathogen: challenges for vaccine development and Progress in the field. J. Infect. Dis. 213, 6–13. doi: 10.1093/infdis/jiv429, 26333944 PMC4676548

[ref123] Post A. S. Guiraud I. Peeters M. Lompo P. Ombelet S. Karama I. . (2022). *Escherichia coli* from urine samples of pregnant women as an indicator for antimicrobial resistance in the community: a field study from rural Burkina Faso. Antimicrob. Resist. Infect. Control 11:112. doi: 10.1186/s13756-022-01142-7, 36064435 PMC9446845

[ref124] Prosser A. Hibbert J. Strunk T. Kok C. H. Simmer K. Richmond P. . (2013). Phagocytosis of neonatal pathogens by peripheral blood neutrophils and monocytes from newborn preterm and term infants. Pediatr. Res. 74, 503–510. doi: 10.1038/pr.2013.145, 23999070

[ref125] Puopolo K. M. Benitz W. E. Zaoutis T. E. (2018). Management of neonates born at ≥35 0/7 weeks' gestation with suspected or proven early-onset bacterial sepsis. Pediatrics 142:2894. doi: 10.1542/peds.2018-2894, 30455342

[ref126] Rad Z. A. Esmaeilzadeh S. Mojaveri M. H. Bagherzadeh M. Javanian M. (2018). Maternal Recto-Vaginal Organisms and Surface Skin Colonization in Infants. Mashhad, Iran: Mashhad University of Medical Sciences.

[ref127] Rashki A. (2014). Cervico-vaginopathogenic *Escherichia coli* in Iran: serogroup distributions, virulence factors and antimicrobial resistance properties. Microb. Pathog. 75, 29–34. doi: 10.1016/j.micpath.2014.08.004, 25193497

[ref128] Raturi A. Chandran S. (2024). Neonatal Sepsis: Aetiology, pathophysiology, diagnostic advances and management strategies. Clin. Med. Insights Pediatr. 18:11795565241281337. doi: 10.1177/11795565241281337, 39371316 PMC11452898

[ref129] Robinson A. E. Heffernan J. R. Henderson J. P. (2018). The iron hand of uropathogenic *Escherichia coli*: the role of transition metal control in virulence. Future Microbiol. 13, 745–756. doi: 10.2217/fmb-2017-0295, 29870278 PMC6161082

[ref130] Russell C. W. Fleming B. A. Jost C. A. Tran A. Stenquist A. T. Wambaugh M. A. . (2018). Context-dependent requirements for FimH and other canonical virulence factors in gut colonization by Extraintestinal pathogenic *Escherichia coli*. Infect. Immun. 86:17. doi: 10.1128/iai.00746-17, 29311232 PMC5820936

[ref131] Russell C. W. Sukumaran R. Liow L. T. Periaswamy B. Rafee S. Chee Y. C. . (2019). Comprehensive identification of Fim-mediated inversions in uropathogenic *Escherichia coli* with structural variation detection using relative entropy. mSphere 4:18. doi: 10.1128/mSphere.00693-18, 30971446 PMC6458436

[ref132] Sáez-López E. Cossa A. Benmessaoud R. Madrid L. Moraleda C. Villanueva S. . (2016a). Characterization of vaginal *Escherichia coli* isolated from pregnant women in two different African sites. PLoS One 11:e0158695. doi: 10.1371/journal.pone.0158695, 27387665 PMC4936694

[ref133] Sáez-López E. Guiral E. Fernández-Orth D. Villanueva S. Goncé A. López M. . (2016b). Vaginal versus obstetric infection *Escherichia coli* isolates among pregnant women: antimicrobial resistance and genetic virulence profile. PLoS One 11:e0146531. doi: 10.1371/journal.pone.0146531, 26784330 PMC4718642

[ref134] Saleh T. Kamau E. Rathe J. A. (2024). New and old lessons from a devastating case of neonatal E coli meningitis. BMC Pediatr. 24:339. doi: 10.1186/s12887-024-04787-y, 38755556 PMC11097427

[ref135] Schrag S. J. Farley M. M. Petit S. Reingold A. Weston E. J. Pondo T. . (2016). Epidemiology of invasive early-onset neonatal Sepsis, 2005 to 2014. Pediatrics 138:2013. doi: 10.1542/peds.2016-2013, 27940705

[ref136] Schulz E. Schumann M. Schneemann M. Dony V. Fromm A. Nagel O. . (2021). *Escherichia coli* Alpha-Hemolysin HlyA induces host cell polarity changes, epithelial barrier dysfunction and cell detachment in human Colon carcinoma Caco-2 cell model via PTEN-dependent dysregulation of cell junctions. Toxins 13:520. doi: 10.3390/toxins13080520, 34437391 PMC8402498

[ref137] Sereme Y. Schrimp C. Faury H. Agapoff M. Lefebvre-Wloszczowski E. Chang Marchand Y. . (2024a). A live attenuated vaccine to prevent severe neonatal *Escherichia coli* K1 infections. Nat. Commun. 15:3021. doi: 10.1038/s41467-024-46775-x, 38589401 PMC11001983

[ref138] Sereme Y. Toumi E. Saifi E. Faury H. Skurnik D. (2024b). Maternal immune factors involved in the prevention or facilitation of neonatal bacterial infections. Cell. Immunol. 396:104796. doi: 10.1016/j.cellimm.2023.104796, 38104514

[ref139] Sherif M. Abera D. Desta K. (2023). Prevalence and antibiotic resistance pattern of bacteria from sepsis suspected neonates at St. Paul's hospital millennium medical college, Addis Ababa, Ethiopia. BMC Pediatr. 23:575. doi: 10.1186/s12887-023-04399-y, 37980512 PMC10656775

[ref140] Shi D. Liu C. Cheng Y. Cheng H. Zhang Q. (2025). Correlation between *Escherichia coli* infection during pregnancy and maternal-fetal outcomes: a retrospective analysis. BMC Infect. Dis. 25:609. doi: 10.1186/s12879-025-10998-0, 40287649 PMC12034207

[ref141] Shimeles G. Gedefie A. Motbainor H. Genet C. (2025). Vaginal colonization, vertical transmission rate, antimicrobial susceptibility profile, and associated factors of potential neonatal pathogens among pregnant women at public health facilities of Northeast Ethiopia. Front. Public Health 13:1475357. doi: 10.3389/fpubh.2025.1475357, 39975790 PMC11836021

[ref142] Silmon de Monerri N. C. Che Y. Lees J. A. Jasti J. Wu H. Griffor M. C. . (2025). Structure-based design of an immunogenic, conformationally stabilized FimH antigen for a urinary tract infection vaccine. PLoS Pathog. 21:e1012325. doi: 10.1371/journal.ppat.1012325, 39970181 PMC12136410

[ref143] Simonsen K. A. Anderson-Berry A. L. Delair S. F. Davies H. D. (2014). Early-onset neonatal sepsis. Clin. Microbiol. Rev. 27, 21–47. doi: 10.1128/cmr.00031-13, 24396135 PMC3910904

[ref144] Šket T. Ramuta T. Starčič Erjavec M. Kreft M. E. (2021). The role of innate immune system in the human amniotic membrane and human amniotic fluid in protection against intra-amniotic infections and inflammation. Front. Immunol. 12:735324. doi: 10.3389/fimmu.2021.735324, 34745106 PMC8566738

[ref145] Snoek L. Gonçalves B. P. Horváth-Puhó E. van Kassel M. N. Procter S. R. Søgaard K. K. . (2022). Short-term and long-term risk of mortality and neurodevelopmental impairments after bacterial meningitis during infancy in children in Denmark and the Netherlands: a nationwide matched cohort study. Lancet Child Adolesc. Health 6, 633–642. doi: 10.1016/s2352-4642(22)00155-9, 35798010 PMC9365703

[ref146] Snoek L. van Kassel M. N. Koelman D. L. H. van der Ende A. van Sorge N. M. Brouwer M. C. . (2023). Recurrent bacterial meningitis in children in the Netherlands: a nationwide surveillance study. BMJ Open 13:e077887. doi: 10.1136/bmjopen-2023-077887, 38159962 PMC10759068

[ref147] Sora V. M. Meroni G. Martino P. A. Soggiu A. Bonizzi L. Zecconi A. (2021). Extraintestinal pathogenic *Escherichia coli*: virulence factors and antibiotic resistance. Pathogens 10:355. doi: 10.3390/pathogens10111355, 34832511 PMC8618662

[ref148] Spaulding C. N. Klein R. D. Ruer S. Kau A. L. Schreiber H. L. Cusumano Z. T. . (2017). Selective depletion of uropathogenic *E. coli* from the gut by a FimH antagonist. Nature 546, 528–532. doi: 10.1038/nature22972, 28614296 PMC5654549

[ref149] Steetskamp J. Zander M. Laufs V. Elger T. Hasenburg A. Skala C. (2024). Does vaginal bacterial colonization contribute to preterm birth in women with asymptomatic shortened cervix? Arch. Gynecol. Obstet. 310, 121–127. doi: 10.1007/s00404-024-07397-2, 38578544 PMC11168983

[ref150] Stocker M. Rosa-Mangeret F. Agyeman P. K. A. McDougall J. Berger C. Giannoni E. (2024). Management of neonates at risk of early onset sepsis: a probability-based approach and recent literature appraisal: update of the Swiss national guideline of the Swiss Society of Neonatology and the pediatric infectious disease group Switzerland. Eur. J. Pediatr. 183, 5517–5529. doi: 10.1007/s00431-024-05811-0, 39417838 PMC11527939

[ref151] Stoll B. J. Puopolo K. M. Hansen N. I. Sánchez P. J. Bell E. F. Carlo W. A. . (2020). Early-onset neonatal Sepsis 2015 to 2017, the rise of Escherichia coli, and the need for novel prevention strategies. JAMA Pediatr. 174:e200593. doi: 10.1001/jamapediatrics.2020.0593, 32364598 PMC7199167

[ref152] Sun H. Li X. Yang X. Qin J. Liu Y. Zheng Y. . (2025). Low leucine levels in the blood enhance the pathogenicity of neonatal meningitis-causing *Escherichia coli*. Nat. Commun. 16:2466. doi: 10.1038/s41467-025-57850-2, 40075077 PMC11904087

[ref153] Tadesse S. Kahsay T. Adhanom G. Kahsu G. Legese H. (2018). Prevalence, antimicrobial susceptibility profile and predictors of asymptomatic bacteriuria among pregnant women in Adigrat general hospital, northern Ethiopia. BMC. Res. Notes 11:740. doi: 10.1186/s13104-018-3844-1 30340646 PMC6194591

[ref154] Tamelienė R. Barčaitė E. Stonienė D. Buinauskienė J. Markūnienė E. Kudrevičienė A. . (2012). *Escherichia coli* colonization in neonates: prevalence, perinatal transmission, antimicrobial susceptibility, and risk factors. Medicina 48, 71–76. doi: 10.3390/medicina48020013, 22491384

[ref155] Tamma P. D. Heil E. L. Justo J. A. Mathers A. J. Satlin M. J. Bonomo R. A. (2024). Infectious Diseases Society of America 2024 guidance on the treatment of antimicrobial-resistant gram-negative infections. Clin. Infect. Dis. doi: 10.1093/cid/ciae403 39108079

[ref156] Tessema B. Lippmann N. Knüpfer M. Sack U. König B. (2021). Antibiotic resistance patterns of bacterial isolates from neonatal Sepsis patients at University Hospital of Leipzig, Germany. Antibiotics 10:323. doi: 10.3390/antibiotics10030323, 33808878 PMC8003699

[ref157] Thomas R. Bijlsma M. W. Gonçalves B. P. Nakwa F. L. Velaphi S. Heath P. T. (2024). Long-term impact of serious neonatal bacterial infections on neurodevelopment. Clin. Microbiol. Infect. 30, 28–37. doi: 10.1016/j.cmi.2023.04.017, 37084940

[ref158] Topcuoglu S. Demirhan S. Dincer E. Ozalkaya E. Karatekin G. (2022). Early-onset neonatal Sepsis in Turkey: A single-center 7-year experience in etiology and antibiotic susceptibility. Children 9:642. doi: 10.3390/children9111642, 36360371 PMC9688980

[ref159] Tseng C. C. Lin W. H. Wu A. B. Wang M. C. Teng C. H. Wu J. J. (2022). *Escherichia coli* FimH adhesins act synergistically with PapGII adhesins for enhancing establishment and maintenance of kidney infection. J. Microbiol. Immunol. Infect. 55, 44–50. doi: 10.1016/j.jmii.2020.09.001, 33023843

[ref160] Tsoumtsa Meda L. L. Landraud L. Petracchini S. Descorps-Declere S. Perthame E. Nahori M. A. . (2022). The cnf1 gene is associated with an expanding *Escherichia coli* ST131 H30Rx/C2 subclade and confers a competitive advantage for gut colonization. Gut Microbes 14:2121577. doi: 10.1080/19490976.2022.2121577, 36154446 PMC9519008

[ref161] van der Flier M. (2021). Neonatal meningitis: small babies, big problem. Lancet Child Adolesc. Health 5, 386–387. doi: 10.1016/s2352-4642(21)00092-4, 33894158

[ref162] Verani J. R. Schrag S. J. (2010). Prevention of perinatal group B streptococcal disease: revised guidelines from CDC. MMWR Recomm. Rep. 59, 1–36. 21088663

[ref163] Viet N. T. Van Du V. Thuan N. D. Van Tong H. Toan N. L. Van Mao C. . (2021). Maternal vaginal colonization and extended-spectrum beta-lactamase-producing bacteria in Vietnamese pregnant women. Antibiotics 10:34067975. doi: 10.3390/antibiotics10050572, 34067975 PMC8152252

[ref164] Vila J. Sáez-López E. Johnson J. R. Romling U. Dobrindt U. Cantón R. . (2016). *Escherichia coli*: an old friend with new tidings. FEMS Microbiol. Rev. 40, 437–463. doi: 10.1093/femsre/fuw005, 28201713

[ref165] Villavicencio-Carrisoza O. Grobeisen-Duque O. Garcia-Correa A. L. Monroy-Muñoz I. E. Villeda-Gabriel G. Sosa-González I. E. . (2025). Advancing understanding of *Escherichia coli* pathogenicity in preterm neonatal Sepsis. Microorganisms. 13:219. doi: 10.3390/microorganisms13020219, 40005586 PMC11857785

[ref166] Vissing N. H. Mønster M. B. Nordly S. Dayani G. K. Heedegaard S. S. Knudsen J. D. . (2021). Relapse of neonatal *Escherichia coli* meningitis: did we miss something at first? Children 8:126. doi: 10.3390/children8020126, 33578792 PMC7916591

[ref167] Wabe Y. A. Reda D. Y. Abreham E. T. Gobene D. B. Ali M. M. (2020). Prevalence of asymptomatic bacteriuria, associated factors and antimicrobial susceptibility profile of bacteria among pregnant women attending Saint Paul's hospital millennium medical college, Addis Ababa, Ethiopia. Ther. Clin. Risk Manag. 16, 923–932. doi: 10.2147/tcrm.S267101, 33061397 PMC7532909

[ref168] Walker M. K. Diao G. Warner S. Babiker A. Neupane M. Strich J. R. . (2024). Carbapenem use in extended-spectrum cephalosporin-resistant Enterobacterales infections in US hospitals and influence of IDSA guidance: a retrospective cohort study. Lancet Infect. Dis. 24, 856–867. doi: 10.1016/s1473-3099(24)00149-x, 38679036 PMC11283355

[ref169] Wang C. Li Q. Lv J. Sun X. Cao Y. Yu K. . (2020). Alpha-hemolysin of uropathogenic *Escherichia coli* induces GM-CSF-mediated acute kidney injury. Mucosal Immunol. 13, 22–33. doi: 10.1038/s41385-019-0225-6, 31719643 PMC6914670

[ref170] Wang S. Ryan C. A. Boyaval P. Dempsey E. M. Ross R. P. Stanton C. (2020). Maternal vertical transmission affecting early-life microbiota development. Trends Microbiol. 28, 28–45. doi: 10.1016/j.tim.2019.07.010, 31492538

[ref171] Wang Z. Xiu X. Zhong L. Wang Y. Fang Z. Lin S. . (2024). Significance of cervical secretion culture in predicting maternal and fetal outcome in pregnant women with premature rupture of membranes: a retrospective cohort study. Front. Pharmacol. 15:1328107. doi: 10.3389/fphar.2024.1328107, 38455965 PMC10917918

[ref172] Waters D. Jawad I. Ahmad A. Lukšić I. Nair H. Zgaga L. . (2011). Aetiology of community-acquired neonatal sepsis in low and middle income countries. J. Glob. Health 1, 154–170. doi: 10.1016/S0140-6736(10)60549-1, 23198116 PMC3484773

[ref173] Watt S. Lanotte P. Mereghetti L. Moulin-Schouleur M. Picard B. Quentin R. (2003). *Escherichia coli* strains from pregnant women and neonates: intraspecies genetic distribution and prevalence of virulence factors. J. Clin. Microbiol. 41, 1929–1935. doi: 10.1128/jcm.41.5.1929-1935.2003, 12734229 PMC154741

[ref174] Wen S. C. H. Ezure Y. Rolley L. Spurling G. Lau C. L. Riaz S. . (2021). Gram-negative neonatal sepsis in low- and lower-middle-income countries and WHO empirical antibiotic recommendations: A systematic review and meta-analysis. PLoS Med. 18:e1003787. doi: 10.1371/journal.pmed.1003787, 34582466 PMC8478175

[ref175] WHO. WHO Bacterial Priority Pathogens List, 2024: Bacterial Pathogens of Public Health Importance to Guide Research, Development and Strategies to Prevent and Control Antimicrobial Resistance. Geneva: WHO (2024). 10.1016/S1473-3099(25)00118-5 PMC12367593 40245910

[ref176] Williams M. Jones A. B. Maxedon A. L. Tabakh J. E. McCloskey C. B. Bard D. E. . (2021). Whole-genome sequencing-based phylogeny, antibiotic resistance, and invasive phenotype of *Escherichia coli* strains colonizing the cervix of women in preterm labor. BMC Microbiol. 21:330. doi: 10.1186/s12866-021-02389-7, 34861816 PMC8641181

[ref177] Williams P. C. Qazi S. A. Agarwal R. Velaphi S. Bielicki J. A. Nambiar S. . (2022). Antibiotics needed to treat multidrug-resistant infections in neonates. Bull. World Health Organ. 100, 797–807. doi: 10.2471/blt.22.288623, 36466207 PMC9706347

[ref178] Wisgrill L. Groschopf A. Herndl E. Sadeghi K. Spittler A. Berger A. . (2016). Reduced TNF-α response in preterm neonates is associated with impaired nonclassic monocyte function. J. Leukoc. Biol. 100, 607–612. doi: 10.1189/jlb.4A0116-001RR, 26965638

[ref179] Wyrsch E. R. Bushell R. N. Marenda M. S. Browning G. F. Djordjevic S. P. (2022). Global phylogeny and F virulence plasmid carriage in pandemic *Escherichia coli* ST1193. Microbiol. Spectrum 10:e0255422. doi: 10.1128/spectrum.02554-22, 36409140 PMC9769970

[ref180] Xia F. Cheng J. Jiang M. Wang Z. Wen Z. Wang M. . (2022). Genomics analysis to identify multiple genetic determinants that drive the global transmission of the pandemic ST95 lineage of Extraintestinal pathogenic *Escherichia coli* (ExPEC). Pathogens 11:489. doi: 10.3390/pathogens11121489, 36558824 PMC9781279

[ref181] Xiao R. Li Y. Liu X. Ding Y. Lai J. Li Y. . (2023). Antibiotic susceptibility of *Escherichia coli* isolated from neonates admitted to neonatal intensive care units across China from 2015 to 2020. Front. Cell. Infect. Microbiol. 13:1183736. doi: 10.3389/fcimb.2023.1183736, 37325509 PMC10267875

[ref182] Xu M. Hu L. Huang H. Wang L. Tan J. Zhang Y. . (2019). Etiology and clinical features of full-term neonatal bacterial meningitis: A multicenter retrospective cohort study. Front. Pediatr. 7:31. doi: 10.3389/fped.2019.00031, 30815433 PMC6381005

[ref183] Yang R. Wang X. Liu H. Chen J. Tan C. Chen H. . (2024). Egr-1 is a key regulator of the blood-brain barrier damage induced by meningitic *Escherichia coli*. Cell Commun. Signal 22:44. doi: 10.1186/s12964-024-01488-y, 38233877 PMC10795328

[ref184] Yin H. Blomberg V. Sun L. Yin C. Sütterlin S. (2025). Virulence potential of ESBL-producing *Escherichia coli* isolated during the perinatal period. Am. J. Perinatol. 42, 822–826. doi: 10.1055/a-2427-9065, 39353616 PMC12020720

[ref185] Zelellw D. A. Dessie G. Worku Mengesha E. Balew Shiferaw M. Mela Merhaba M. Emishaw S. (2021). A systemic review and meta-analysis of the leading pathogens causing neonatal Sepsis in developing countries. Biomed. Res. Int. 2021:6626983. doi: 10.1155/2021/6626983, 34195273 PMC8203353

[ref186] Zhang D. Ge X. Wang Y. Gao W. Ding Y. Zhang J. (2025). The possibility of *Escherichia coli* transmission from pregnant women to the neonates. Eur. J. Clin. Microbiol. Infect. Dis. 44, 2919–2927. doi: 10.1007/s10096-025-05257-8, 40965615

[ref187] Zheng N. Guo R. Wang J. Zhou W. Ling Z. (2021). Contribution of *Lactobacillus iners* to vaginal health and diseases: A systematic review. Front. Cell. Infect. Microbiol. 11:792787. doi: 10.3389/fcimb.2021.792787, 34881196 PMC8645935

[ref188] Zheng Y. Sun H. Wang Y. Jin C. Li X. Pang Y. . (2024). CsiR-mediated signal transduction pathway in response to low Iron conditions promotes *Escherichia coli* K1 invasion and penetration of the blood-brain barrier. J. Infect. Dis. 230, e807–e817. doi: 10.1093/infdis/jiae157, 38531686 PMC11481304

